# New Chromone Analogues as Potential Bioactive Compounds: Cyclodextrin Complexation and Molecular Docking

**DOI:** 10.3390/molecules31213990

**Published:** 2026-11-05

**Authors:** Patryk Czapnik, Magdalena Małecka, Pawel Hikisz, Adam Buczkowski, Artur Stepniak, Khalida Khalil, Rehana Bano, Elzbieta Budzisz, Angelika A. Adamus-Grabicka

**Affiliations:** 1 Department of Physical Chemistry, Faculty of Chemistry, University of Lodz, Pomorska 163/165, 90-236 Lodz, Poland; patryk.czapnik@edu.uni.lodz.pl (P.C.); magdalena.malecka@chemia.uni.lodz.pl (M.M.); adam.buczkowski@chemia.uni.lodz.pl (A.B.); artur.stepniak@chemia.uni.lodz.pl (A.S.); khalida.khalil@edu.uni.lodz.pl (K.K.); 2 Doctoral School of Exact and Natural Sciences, University of Lodz, Jana Matejki 21/23, 90-237 Lodz, Poland; 3 Department of Oncobiology and Epigenetics, Institute of Biophysics, Faculty of Biology and Environmental Protection, University of Lodz, Pomorska 141/143, 90-236 Lodz, Poland; pawel.hikisz@biol.uni.lodz.pl; 4 Higher Education and Industrial Cooperation Centre, University of Miskolc, Miskolc-Egyetemváros, 3515 Miskolc, Hungary; rehana.bano@uni-miskolc.hu; 5 Department of the Chemistry of Cosmetic Raw Materials, Faculty of Pharmacy, Medical University of Lodz, Muszynskiego 1, 90-151 Lodz, Poland; elzbieta.budzisz@umed.lodz.pl; 6 Department of Medical Chemistry, Faculty of Pharmacy, Medical University of Lodz, Muszynskiego 1, 90-151 Lodz, Poland

**Keywords:** chromone analogues, cyclodextrins, imidazoles, pyrazoles, inclusion complexes, crystal structure, crystal packing, hydrogen bonds, molecular docking

## Abstract

Chromone is an important structural motif in numerous biologically active natural and synthetic compounds. The aim of the present study is to synthesize and characterize a series of novel chromone derivatives bearing imidazole and pyrazole moieties. The synthesis involved a two-step procedure in which methyl 3-chromonecarboxylate derivatives were prepared and then condensed with imidazole and pyrazole analogues. The structures of the compounds were characterized using spectroscopy, single-crystal X-ray diffraction and Hirshfeld surface analysis. Compounds **1** and **1A** were subjected to ITC titration in DMSO. Anticancer potential of synthetized substances was evaluated against three breast cancer cell lines (MCF-7, MDA-MB-231 and HCC38), and two endometrial cancer cell lines (Ishikawa and Hec-1-A). Compounds **1B** and **1C**, bearing 3-(1*H*-imidazol-1-yl)propan-1-amine and3-(3,5-dimethyl-4*H*-pyrazol-4-yl)propan-1-amine moieties, respectively, exhibited the most pronounced, albeit modest, cytotoxic activity against MCF-7 cells, with IC_50_ values of approximately 80 μM. In HMEC-1 human microvascular endothelial cells (used as a non-cancerous reference model), the same compounds lowered the signal of the dihydroethidium (DHE) probe, suggesting a modest antioxidant effect; these redox results are reported separately from the anticancer assays. The compounds were subjected to further complex analysis using 2′,7′-dichlorodihydrofluorescein diacetate (H_2_DCFDA) and molecular docking studies. This complex approach provided a qualitative insight into the structure–activity relationships of the studied derivatives. Given the modest cytotoxicity and limited selectivity of Compounds **1B** and **1C**, together with their redox-modulating effect observed in HMEC-1 cells, these compounds should be regarded as a starting point for further investigation rather than as promising anticancer candidates.

## 1. Introduction

Chromone (4*H*-1-benzopiran-4-on) is a privileged heterocyclic scaffold in medicinal chemistry and represents an important structural motif in numerous biologically active natural and synthetic compounds. Its relatively simple core enables extensive structural modification, resulting in derivatives with diverse pharmacological properties, including anticancer, anti-inflammatory, antimicrobial, antioxidant, antidiabetic and neuroprotective activities. Importantly, the nature and position of substituents on the chromone scaffold can substantially influence its biological activity and molecular interactions with biological targets, making chromone a valuable platform for the design of new bioactive compounds [1,2,3].

One important tool for studying chromone derivatives is molecular docking, which has proven to be of significant value for elucidating their possible interactions with biologically relevant molecular targets. By predicting ligand-binding modes and identifying potential non-covalent interactions within target binding sites, docking can provide a molecular-level rationale for observed or expected biological activity and support the structure-based development of new chromone analogues [4]. Thus, molecular docking may be particularly useful for relating structural modifications of newly synthesized chromones to their potential biological targets.

The pharmaceutical potential of bioactive chromone derivatives can be improved by complexation with cyclodextrins (CDs). The formation of host–guest inclusion complexes may improve the solubility, stability and bioavailability of the guest compounds [5,6].

Molecular docking can complement experimental studies by providing information about the preferred orientation of a chromone derivative within the cyclodextrin cavity and the intermolecular interactions contributing to complex stabilization. Such computational analysis has been increasingly used to elucidate the molecular mechanisms of cyclodextrin–guest recognition and to rationalize experimentally observed inclusion behaviour [7]. While molecular modelling provides a structural insight into host–guest recognition, experimental determination of the thermodynamics of complex formation is essential for a comprehensive characterization of these systems.

Isothermal titration calorimetry (ITC) enables direct determination of the binding constant (Ka), proposed stoichiometry (n), and enthalpy change (ΔH) of cyclodextrin–guest interactions, when the heat exchange during titration is high or moderate. In the case of reactions occurring with very low heat exchange, these binding parameters are not always possible to calculate. From determined binding constant (Ka), and enthalpy change (ΔH) further thermodynamic functions like the corresponding Gibbs free energy (ΔG) and entropy contribution (ΔS) can also be calculated. Therefore, if possible, combining ITC with molecular docking provides information about the impact of solvent on the molecular basis of inclusion complex formation [8,9].

The aim of this study was to investigate the biological potential of newly synthesized chromone analogues condensed with pyrazole and imidazole rings, with particular emphasis on their antioxidant activity, cytotoxicity against selected cancer cell lines, and molecular interactions with β-cyclodextrin. In the present study, new chromone analogues condensed with pyrazole and imidazole were synthesized and investigated using a combination of experimental and computational approaches. Their potential host–guest interactions with β-cyclodextrins were investigated by molecular docking. This integrated approach was used to establish relationships between the chemical structure of the chromone analogues, their predicted interactions with biological targets, and their inclusion modes within the cyclodextrin cavity. Based on computational studies, assays were performed to evaluate antioxidant activity and cytotoxicity against selected cancer cell lines. The results provide insight into both the biological potential of the newly synthesized chromone derivatives and the molecular basis of their interactions with cyclodextrins.

## 2. Results and Discussion

### 2.1. Structural Analysis

**Figure 1:**
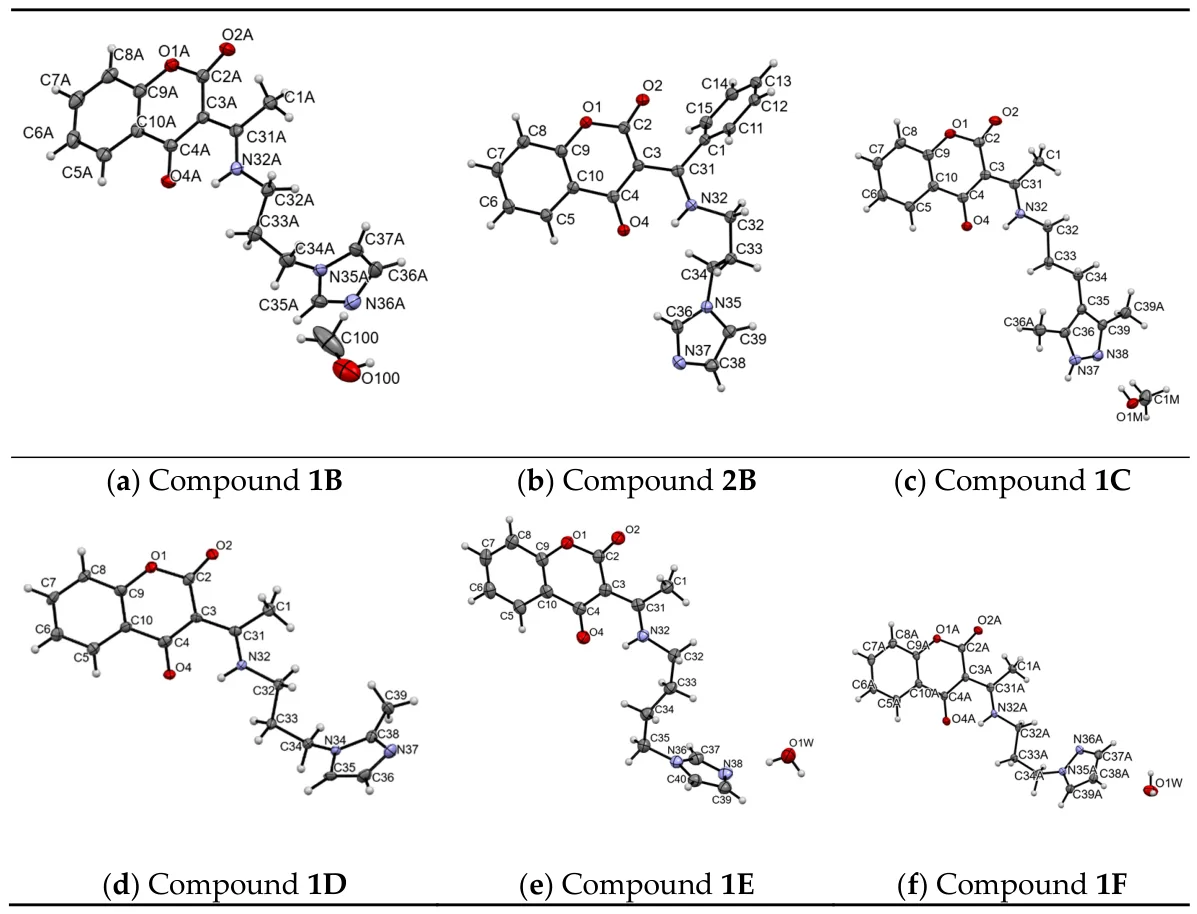
Molecular structures of all synthesized compounds with atoms numbering. For Compound **1B** and Compound **1F,** only one of two molecules in asymmetric unit is presented.

For all synthesized compounds, crystal structures were determined using X-ray studies. Crystallographic data and detailed structural characteristics of six selected compounds are collected in **Table 1**. The molecular structures of all synthesized chromone analogues (Compounds **1B–1F**, **2B**) are presented in **Figure 1**. Compounds **1B** and **1F** comprise two chromone analogue molecules in an asymmetric unit. The crystal lattices may also include a methanol molecule (in Compound **1C**) and water molecules (in Compounds **1E** and **1F**).

All studied compounds are structurally similar to previously published analogues (compounds **III** and **IV** in [10]). All determined compounds are composed of a flat benzopyran skeleton to which an oxygen atom is attached in positions 2 and 4. However, they differ regarding the substituent in position 3, where the following imidazole and pyrazole derivatives are attached to the carbon atom:

- 3-(1*H*-imidazol-1-yl)propan-1-amine (Compounds **1B** and **2B**).
- 3-(3,5-dimethyl-4*H*-pyrazol-4-yl)propan-1-amine (Compound **1C**).
- 3-(2-methyl-1*H*-imidazol-1-yl)propan-1-amine (Compound **1D**).
- 4-(1*H*-imidazol-1-yl)butan-1-amine (Compound **1E**).
- 3-(1*H*-pyrazol-1-yl)propan-1-amine (Compound **1F**).

**Table 1:** Crystallographic data and details of the structure determination.

	Compound **1B**	Compound **2B**	Compound **1C**	Compound **1D**	Compound **1E**	Compound **1F**
**Crystal data**						
Chemical formula	4(C_17_H_17_N_3_O_3_)·CH_4_O	C_22_H_19_N_3_O_3_	C_19_H_21_N_3_O_3_ ·CH_4_O	C_18_H_19_N_3_O_3_	C_18_H_19_N_3_O_3_ ·H_2_O	2(C_17_H_17_N_3_O_3_)·H_2_O
M_r_	1277.38	373.40	371.43	325.36	343.38	640.69
Crystal system, space group	Monoclinic, P2_1_/n	Monoclinic, P2_1_/n	Monoclinic, P2_1_/c	Triclinic, P-1	Monoclinic, P2_1_/c	Monoclinic, P2_1_/n
Temperature (K)	100	100	100	103	102	101
a, b, c (Å)	7.1000 (1), 14.3869 (2), 29.9636 (4)	7.2321 (1), 17.3639 (1), 14.3372 (1)	18.7234 (3), 10.8219 (1), 9.5951 (1)	7.5406 (2), 9.6673 (3), 11.9719 (4)	8.1480 (2), 9.4039 (2), 22.2714 (4)	10.9688 (1), 20.1720 (2), 14.0055 (1)
α, β, γ (°)	94.336 (1)	92.154 (1)	104.637 (1)	α = 84.654 (2), β = 82.391 (2), γ = 68.335 (3)	98.422 (2)	101.555 (1)
V (Å^3^)	3051.93 (7)	1799.16 (3)	1881.09 (4)	803.00 (5)	1688.10 (6)	3036.09 (5)
Z	2	4	4	2	4	4
F(000)	1348	784	792	344	728	1352
Radiation type	Cu Kα	Cu Kα	Cu Kα	Cu Kα	Cu Kα	Cu Kα
No. of reflections for cell measurement	13,835	18,302	10,417	16,572	14,293	28,142
θ range (°) for cell measurement	3.0–76.1	4.0–76.7	4.7–77.1	3.7–76.7	4.0–75.1	3.9–76.4
µ (mm^−1^)	0.81	0.76	0.76	0.76	0.80	0.82
Crystal size (mm)	0.45 × 0.05 × 0.03	0.27 × 0.21 × 0.07	0.29 × 0.08 × 0.06	0.18 × 0.07 × 0.03	0.18 × 0.11 × 0.06	0.20 × 0.14 × 0.07
**Data collection**						
Diffractometer	XtaLAB Synergy, Dualflex, HyPix	XtaLAB Synergy, Dualflex, HyPix	XtaLAB Synergy, Dualflex, HyPix	XtaLAB Synergy, Dualflex, HyPix	XtaLAB Synergy, Dualflex, HyPix	XtaLAB Synergy, Dualflex, HyPix
Radiation source	micro-focus sealed X-ray tube	micro-focus sealed X-ray tube	micro-focus sealed X-ray tube	micro-focus sealed X-ray tube,	micro-focus sealed X-ray tube,	micro-focus sealed X-ray tube
Detector resolution (pixels mm^−1^)	10.0000	10.0000	10.0000	10.0000	10.0000	10.0000
Absorption correction	Multi-scan *	Multi-scan *	Multi-scan *	Multi-scan *	Multi-scan *	Multi-scan *
T_min_, T_max_	0.487, 1.000	0.866, 1.000	0.663, 1.000	0.713, 1.000	0.873, 1.000	0.633, 1.000
No. of measured, independent and observed [I > 2σ(I)] reflections	26,462, 5490, 4834	27,166, 3591, 3367	14,718, 3260, 3002	22,738, 2880, 2768	24,181, 3034, 2787	45,971, 6198, 5896
R_int_	0.062	0.025	0.032	0.036	0.047	0.049
θ values (°)	θ_max_ = 67.5, θ_min_ = 3.0	θ_max_ = 77.1, θ_min_ = 4.0	θ_max_ = 67.5, θ_min_ = 4.8	θ_max_ = 67.4, θ_min_ = 3.7	θ_max_ = 67.5, θ_min_ = 4.0	θ_max_ = 77.1, θ_min_ = 3.9
(sin θ/λ)_max_ (Å^−1^)	0.599	0.632	0.599	0.599	0.599	0.632
**Refinement**						
Refinement on	F^2^	F^2^	F^2^	F^2^	F^2^	F^2^
R[F^2^ > 2σ(F^2^)], wR(F^2^), S	0.074, 0.178, 1.09	0.033, 0.088, 1.05	0.040, 0.108, 1.04	0.120, 0.363, 1.16	0.038, 0.095, 1.03	0.095, 0.259, 1.17
No. of reflections	5490	3591	3260	2880	3034	6198
No. of parameters	437	253	249	219	230	426
No. of restraints	12	0	0	0	0	0
H-atom treatment	H-atom parameters constrained	H-atom parameters constrained	H-atom parameters constrained	H-atom parameters constrained	H-atom parameters constrained	H-atom parameters constrained
Δρ_max_, Δρ_min_ (e Å^−3^)	0.74, −0.43	0.24, −0.22	0.36, −0.35	0.79, −0.58	0.19, −0.22	0.80, −0.49

Computer programs: *CrysAlis PRO* 1.171.44.109a [11], SHELXT 2018/2 [12], SHELXL 2018/3 [13], Olex2 1.5 [14]. * Empirical absorption correction using spherical harmonics, implemented in SCALE3 ABSPACK scaling algorithm. All crystal structures are stabilized by numerous inter- and intramolecular hydrogen bonds. Detailed analysis is included in SI in **Tables S1–S7 (Supplementary Material) and Figures S1–S15**.

### 2.2. Hirshfeld Surface (HS) Analysis

**Figure 2:**
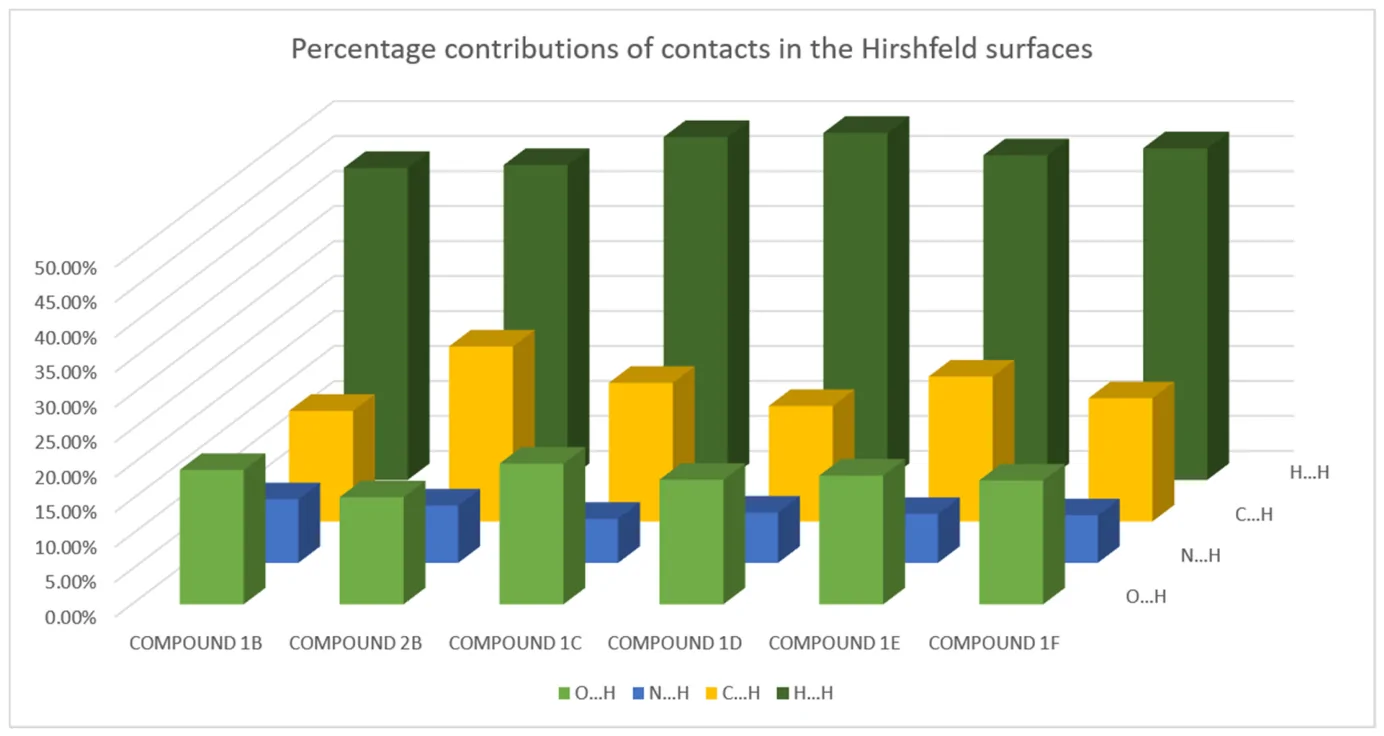
Percentage contribution of contacts in the Hirshfeld surfaces of all chromone analogues determined by X-ray diffraction in this work.

For the six new chromone derivatives, Hirshfeld surfaces (HS) and fingerprint plots of the determined crystal structures were generated to identify the percentage contribution of contacts in the HS. Contacts with the highest percentage contributions in the HS are presented in **Figure 2**. For all chromone analogues, the largest percentage share in the HS of all chromone analogues is observed for H…H contacts and is in the range from 44.60% to 49.60%. Additionally, a significant role was played by O…H contacts in four compounds (Compounds **1B**, **1C**_,_ **1D** and **1E**) and by C…H contacts in two (Compounds **1E and 2B**). The contribution of N…H contacts did not exceed 9.10% (6.30–9.10%).

### 2.3. Isothermal Titration Calorimetry (ITC)

**Figure 3:**
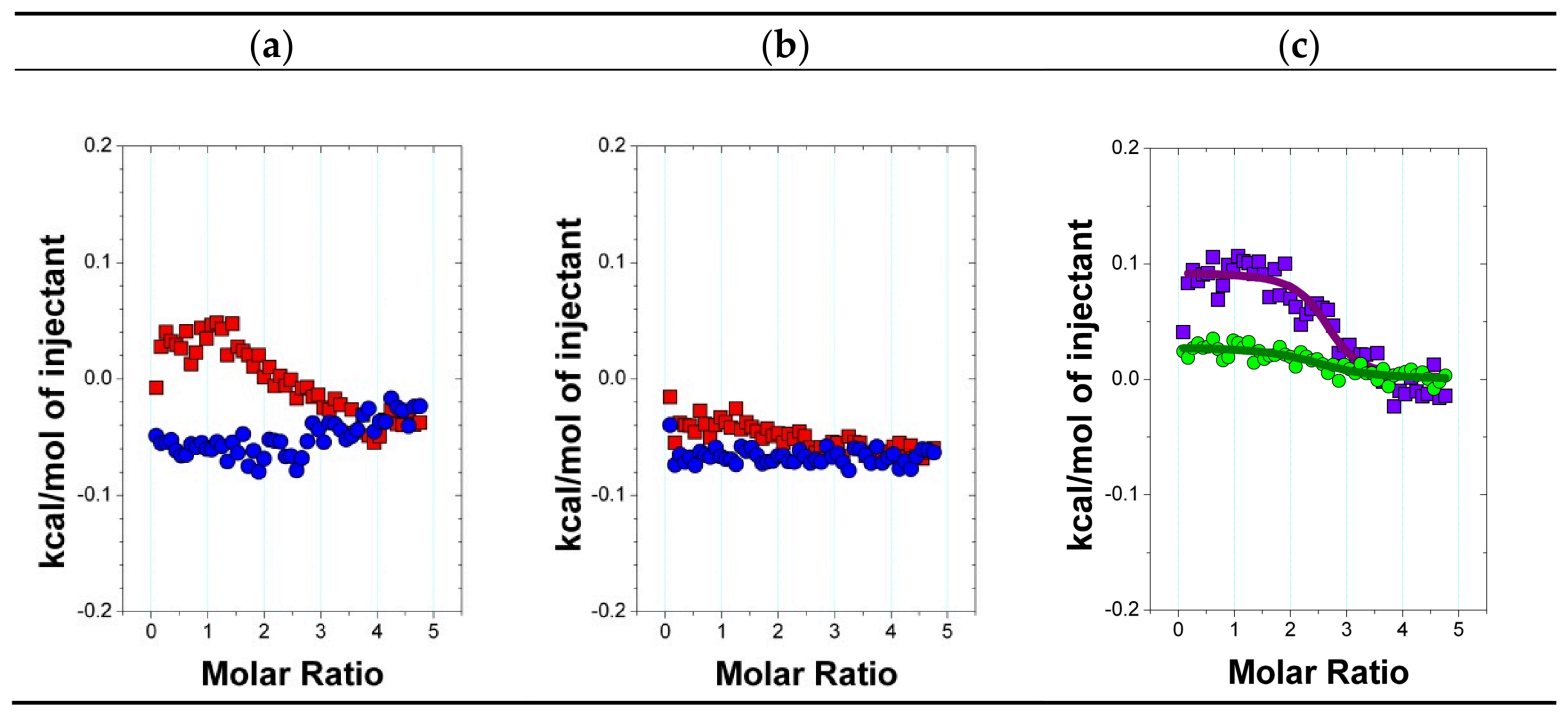
Heat effects of: (**a**) ITC titration of 0.4 mM Compound **1** by 10 mM β-CD solution (■) in DMSO as a function of molar ratio of β-CD to Compound **1** and corresponding effects of β-CD dilution (●), (**b**) ITC titration of 0.4 mM Compound **1A** by 10 mM β-CD solution (■) in DMSO as a function of molar ratio of β-CD to Compound **1A** and corresponding effects of β-CD dilution (●), (**c**) direct interactions between chromone derivative Compound **1** (■) and Compound **1A** (●) with β-CD in DMSO as a function of molar ratio of β-CD to chromone derivative. The solid lines represent the fitting curve calculated with the one set of binding site model.

To assess the interaction of the selected chromone analogues with β-cyclodextrin (β-CD), the compounds were subjected to isothermal titration calorimetry in DMSO, using a VP-ITC microcalorimeter (MicroCal, USA). A solution of 0.4 mM chromone analogue was titrated with β-CD solution at 25 °C (**Figure 3a,b**). After accounting for the thermal effects of β-CD dilution, the thermal effects of direct interaction between chromone analogue and β-CD (**Figure 3c**) were fitted. The ITC titrations were performed for all new chromones; however, for Compound **1** (substrate) and the additional reference Compound **1A**, it was possible to determine various complex apparent stoichiometry (n) and binding parameters (**Table 2**), *viz.* binding constant (K), as well as enthalpy (Δ*H*), entropy (Δ*S*), and Gibbs free energy change (Δ*G*), by using a fitting procedure with one set of binding site.

According to the obtained ITC results (**Table 2**), the values of the fitted stoichiometric parameter *n* = 2.6 for both Compound **1** and Compound **1A** indicate that in the mixture with a high molar content of β-CD relative to these guests, complexes (associates) with apparent higher stoichiometry are formed, in which one chromone derivative molecule interacts with at least two macrocycles of β-CD. Complexation of both Compound **1** and Compound **1A** with β-CD is thermodynamically spontaneous (∆G < 0) and endothermal (∆H > 0) in DMSO at 25 °C. For both derivatives, their enthalpy of binding with β-CD is endothermic (ΔH > 0), indicating that the partial desolvation effects of the host (β-CD) and guest (chromone derivatives) functional groups predominate over those of their direct interactions. For both derivatives, their interaction with β-CD is governed predominantly by the increase in the disordering state of reagents (∆S > 0), which overcomes the endothermal enthalpy of interactions.

Both Compound **1** and Compound **1A** exhibit high apparent binding constants (logK_a_ > 4) to β-CD, which confirms the thermodynamic spontaneity of interactions between components of the complex. Compound **1** demonstrates stronger binding interactions with β-CD in DMSO at 25 °C compared to the more sterically developed derivative **1A**: Compound **1** exhibits a higher binding constant and higher entropy of interaction with β-CD than Compound **1A**.

### 2.4. Molecular Docking

**Figure 4:**
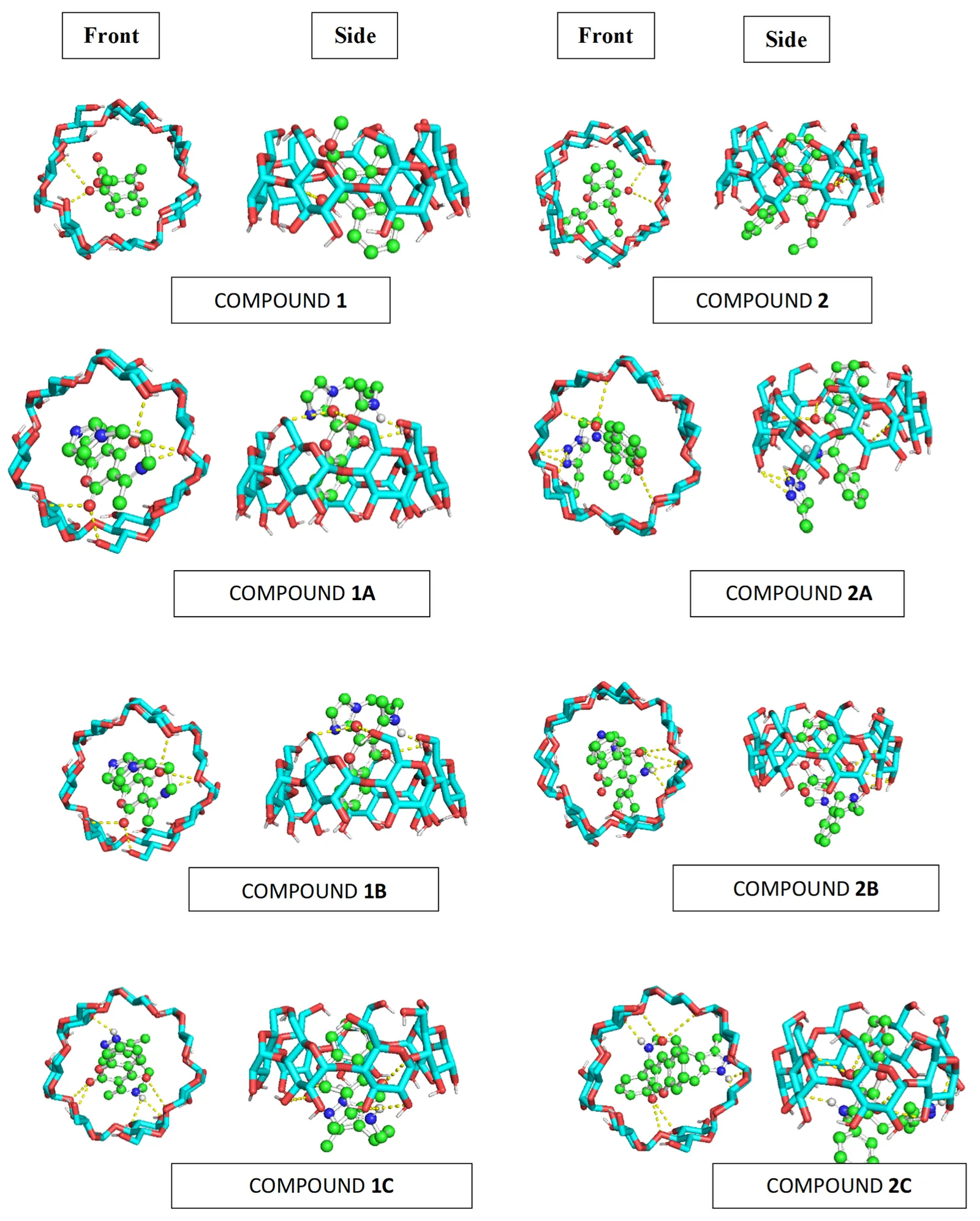

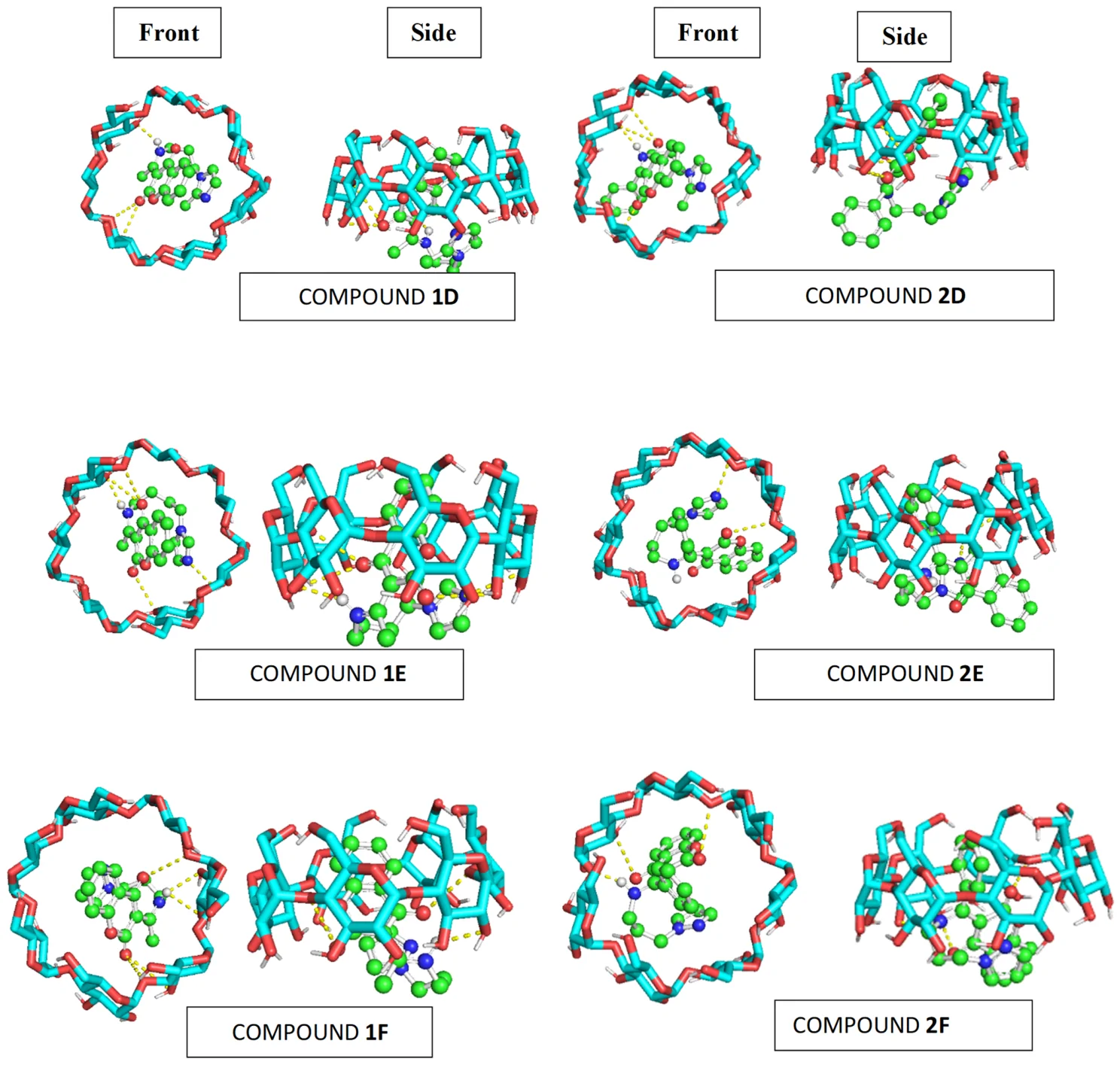
3D structures of chromone derivatives complexed with β-CD.

The host–guest interactions between β-cyclodextrin and the chromone derivatives were evaluated using molecular docking. The calculated binding energies ranged from −5.3 to −6.6 kcal/mol, indicating favourable interactions between the guests and the cyclodextrin cavity. According to **Table 3**, Compound **2C** exhibited the most favorable predicted binding energy (−6.6 kcal/mol), followed by Compound **1A** (−6.4 kcal/mol) and Compound **1C** (−6.3 kcal/mol). The front- and side-view docking poses (**Figure 4**) show that the guest molecules are accommodated within the cyclodextrin cavity or partially inserted within; this supports the data regarding the potential additional β-CDs involved in the complexation, especially in Compound **1** and Compound **1A** (Section 3.3).

Several complexes exhibit multiple close contacts and potential hydrogen-bonding interactions (**Figure 4**, indicated by dashed lines). The aromatic/hydrophobic regions of the chromone derivatives can interact with the relatively hydrophobic interior of the cyclodextrin cavity, while polar functional groups may form additional interactions near the hydroxyl-rich rim.

The three highest-ranked poses generally showed only small energy differences (0.1–0.3 kcal/mol), suggesting that closely related binding orientations are energetically favourable. For example, Compound **1D** showed −6.4, −6.3, and −6.3 kcal/mol, while Compound **2C** exhibited −6.6, −6.5, and −6.3 kcal/mol for the top three poses. Of all the tested compounds, **2C/β-CD** exhibited the strongest predicted host–guest interactions. Overall, the docking results indicate that the investigated derivatives can form favourable and relatively stable inclusion complexes with β-cyclodextrin. This is consistent with the favorable complex formation indicated by the ITC results. However, it has to be noted that the docking scores represent qualitative preference rather than exact thermodynamic properties. The molecular docking calculations presented in this study examine individual 1:1 host–guest pairs and provide complementary information about possible binding orientations and local intermolecular interactions, rather than direct structural validation of the higher-order assemblies.

Predicted molecular docking poses of the chromone derivatives within β-cyclodextrin (β-CD), shown in front and side views. The representations illustrate the predicted orientation and degree of insertion of each guest molecule into the β-CD cavity. Selected close-up views highlight the principal predicted non-covalent interactions, including hydrogen-bonding contacts and other relevant close contacts where identified by geometric analysis. Dashed lines indicate the interactions annotated in the corresponding panels. The depicted structures represent computational predictions for individual 1:1 host–guest complexes and do not establish the stoichiometry or complete structure of the higher-order assemblies suggested by ITC.

### 2.5. Biological Assay

#### 2.5.1. Determination of Cell Viability by Metabolic Microplate Spectrophotometric Assay with MTT

The in vitro cytotoxicity of the chromone analogues was assessed by MTT assay against three breast cancer cell lines (MCF7, MDA-MB-231, HCC38) and two endometrial cancer cell lines (Ishikawa, Hec-1A); these were supplemented with the immortalized HMEC-1 human microvascular endothelial line as a normal cell model (**Table 4**). Cells were exposed to the compounds for 24 h over a wide concentration range (0–120 µM); IC_50_ values were calculated as the concentration required to reduce the fraction of surviving cells to 50% of the non-treated control. The results are given as the mean ± SEM of three independent experiments.

All six analogues exhibited high IC_50_ values indicating low to marginal intrinsic cytotoxicity in every cell line. The most active analogues were Compound **1B** and Compound **1C**, which produced measurable growth inhibition in several lines, with IC_50_ values in the range of 81–119 µM. Specifically, Compound **1B** exhibited IC_50_ values of 81.2 ± 1.6, 91.5 ± 1.9 µM (MDA-MB-231), 118.2 ± 2.2 µM (HCC38) and 114.8 ± 4.9 µM (Hec-1A); in addition, IC_50_ values of >120 µM were observed against Ishikawa cells and 119.1 ± 1.4 µM against HMEC-1.

Similarly, Compound **1C** demonstrated IC_50_ values of 82.5 ± 2.1 µM (MCF7), 87.0 ± 3.2 µM (MDA-MB-231), 116.9 ± 2.8 µM (HCC38) and 117.9 ± 6.5 µM (Hec-1A); it similarly demonstrated values > 120 µM against Ishikawa cells; however, exhibited the lowest IC_50_ value against the HMEC-1 line (102.5 ± 2.1 µM). The corresponding selectivity indices (SI = IC_50_(HMEC-1)/IC_50_(cancer cell line); Table 4) were modest: for Compound **1B**, SI = 1.47 (MCF7), 1.30 (MDA-MB-231), 1.01 (HCC38) and 1.04 (Hec-1A), whereas in Ishikawa cells the SI could not be determined because the IC_50_ exceeded the tested concentration range (SI < 0.99); for Compound 1C, SI = 1.24 (MCF7), 1.18 (MDA-MB-231), 0.88 (HCC38) and 0.87 (Hec-1A), with SI < 0.85 in Ishikawa cells. These values confirm that the in vitro selectivity window of the two compounds is narrow. The remaining analogues were essentially inactive within the tested concentration range: Compound **1D** produced no measurable inhibition in any cell line (IC_50_ > 120 µM throughout), Compound **1E** showed a cytotoxic effect only against MCF7 (113.4 ± 2.7 µM) and, marginally, HCC38 (119.8 ± 4.6 µM) Compound **1F** was active in MCF7 (114.1 ± 5.9 µM) and Ishikawa (119.6 ± 5.1 µM), whereas in MDA-MB-231 the IC_50_ value exceeded the upper tested concentration (>120 µM), and Compound **2B** showed detectable activity only against Ishikawa (118.2 ± 2.7 µM).

The activity of **1B** and **1C** is similar to values reported for chromone and flavonoid scaffolds with comparable substitution complexity. The parent flavonol, quercetin, an archetypal chromone-derived natural product, inhibits the proliferation of MCF-7 cells with IC_50_ values ranging from 23 to 73 µM, depending on the study [15]. While related flavonoid–chromone systems act in a similar concentration range, more extensively modified chromone hybrids, such as carbazolyl–thiazolyl chromone–pyrazole systems, exhibit low-micromolar IC_50_ values [16]. The IC_50_ values of 81–92 µM obtained here for **1B** and **1C** were determined after a 24 h exposure and characterize the activity of the compounds under the applied protocol; they should be compared with literature data obtained under longer exposure times (48–72 h) with caution and correspond to the moderate cytotoxicity typical of mono-substituted chromone derivatives. The results indicate that the aminopropyl-imidazole/pyrazole motif is a starting point for further structural optimization; however, given the modest potency and the limited selectivity indices (SI ≤ 1.47), Compounds **1B** and **1C** should be regarded as preliminary hits for further development rather than as promising anticancer candidates. Notably, the activity profiles of **1B** and **1C** are broad: both compounds inhibit the proliferation of the oestrogen-receptor-positive MCF-7 line. More importantly, they also inhibit the triple-negative MDA-MB-231 line (IC_50_ ≈ 87–92 µM), which is characterized by high therapeutic resistance and few targeted treatment options.

The structure–activity relationship within the series is clear. The key activity-bearing elements are the 3-(1*H*-imidazol-1-yl)propan-1-amine (**1B**) and 3-(3,5-dimethyl-4*H*-pyrazol-4-yl)propan-1-amine (**1C**) side chains. The remaining modifications, *viz.* methylation at the 2-position of the imidazole ring (**1D**), elongation of the spacer to a butyl chain (**1E**), replacement of imidazole by pyrazole (**1F**) and enlargement of the ester group (**2B**), markedly reduce the cytotoxicity, providing a well-defined direction for lead optimization. An equally important role is also played by the tolerability signal of the series: the less active derivatives **1D**, **1E**, **1F** and **2B** appear to be essentially non-toxic towards the non-cancerous HMEC-1 line (IC_50_ > 120 µM), and even the most active compound, **1B**, retained a measurable preference for cancer cells over HMEC-1 (81.2 µM vs 119.1 µM; SI = 1.47), although this preference is only moderate and does not guarantee a sufficient therapeutic window.

#### 2.5.2. Determination of Antioxidant Potential Using DHE and H_2_DCFDA Fluorescent Probes

**Figure 5:**
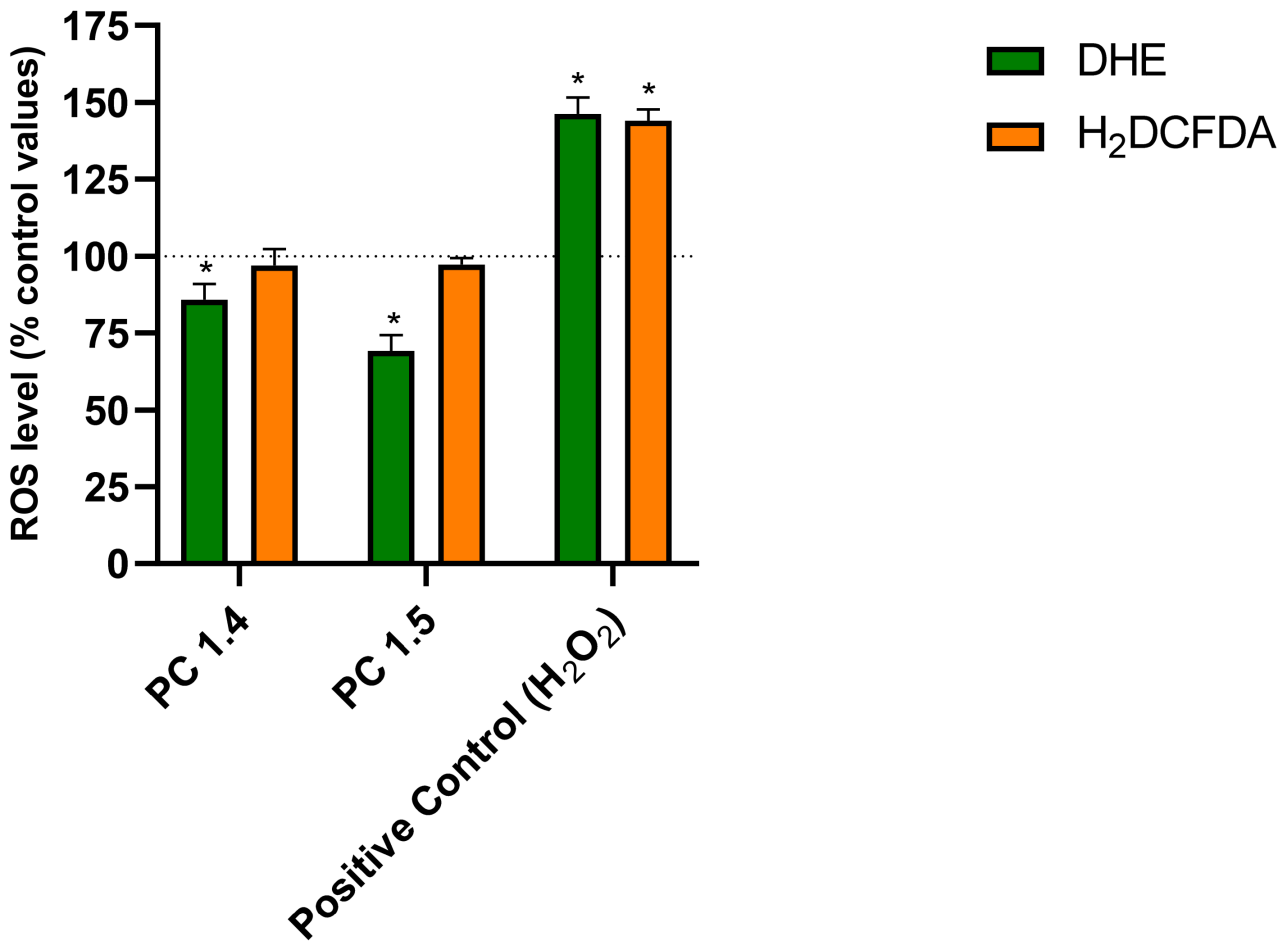
Intracellular ROS levels in human dermal microvascular endothelial cells (HMEC-1) following exposure to the chromone derivatives **Compound 1B** and **Compound 1C**. Cells were incubated for 24 h with 50 µmol/l of the tested compounds; hydrogen peroxide 50 µmol/l served as the positive control and non-treated cells as the reference (dashed line, 100%). The fluorescence derived from the DHE probe, used here as an overall indicator of the oxidation processes reported by this probe, was monitored, while the overall redox-sensitive intracellular signal was assessed with H_2_DCFDA; results are expressed as the percentage of the control fluorescence. Bars represent the mean ± SD of n = 3 independent experiments. * *p* < 0.05 compared with the control one-way ANOVA with Dunnett’s *post hoc* test.

The redox properties of the two most active derivatives, compounds **1B** and **1C**, were evaluated in HMEC–1 cells using two complementary fluorescent probes: dihydroethidium (DHE), the oxidation of which yields several fluorescent products commonly used as an indicator of cellular oxidation processes, although DHE fluorescence alone does not specifically quantify superoxide, as several fluorescent oxidation products can be formed [17,18], and 2′,7′–dichlorodihydrofluorescein diacetate (H_2_DCFDA), which reports an overall redox-sensitive intracellular signal rather than a selective measure of hydrogen peroxide [17,19]. The cells were preincubated for 24 h with the test compounds (50 µM) or vehicle. Positive controls were set up by exposure to 50 µM H_2_O_2_. In all cells, the fluorescence was quantified relative to untreated controls (**Figure 5**). As expected, the oxidative challenge induced a ~145% response in the DHE test and ~143% for H_2_DCFDA, confirming the responsiveness of the system.

In the H_2_O_2_-challenged cells, **1B** reduced the DHE-derived signal to approximately 85% of the untreated, non-challenged control and **1C** to approximately 69% (*p* < 0.05, one-way ANOVA with Dunnett’s post hoc test). In contrast, neither compound significantly altered the H_2_DCFDA signal (96% and 97% of the untreated control, respectively), and no increase in probe fluorescence was observed in any of the compound-treated groups; hence, pro-oxidant redox cycling is unlikely to occur.

The effect is therefore notable for its directionality: both derivatives lowered the DHE-derived signal below the constitutive baseline without changing the DCF readout. However, because neither probe is species-specific, these observations indicate a shift in the redox processes reported by the two probes, but do not allow the identity of the reactive species involved or the molecular target of the compounds to be determined.

The interpretation of these findings requires caution. Although the reaction of DHE with superoxide yields 2-hydroxyethidium as one of the products [17,18], DHE can also undergo oxidation to several other fluorescent species (e.g., ethidium and its dimers), so the total DHE-derived signal cannot be regarded as a specific quantitative measure of superoxide. Similarly, H_2_DCFDA reports an overall redox-sensitive signal rather than hydrogen peroxide alone [17,19]. The observed, below-baseline reduction of the DHE-derived signal with an unchanged H_2_DCFDA readout may therefore suggest that the processes reported by DHE are affected by **1B** and **1C** without a compensatory increase in the signal reported by H_2_DCFDA; however, the present experiments do not allow any conclusion as to the identity of the reactive species involved or the underlying molecular mechanism, and hypotheses such as scavenging of superoxide, interference with superoxide-generating systems (e.g., NADPH oxidase or mitochondrial electron leakage) or other redox mechanisms remain speculative and require dedicated experiments. Redox activity of chromone-derived systems is well precedented, exemplified by a chromone-based copper(II) complex displaying high superoxide dismutase activity [20], and the superoxide-scavenging capacity of flavonoid chromone derivatives has been reported [15]. Importantly, no increase in probe fluorescence was detected in any treatment group, arguing against pro-oxidant redox cycling—a valuable safety attribute for redox-active pharmacophores.

The biological implications of these observations should be interpreted with caution. In non-cancerous endothelial cells, superoxide is a key mediator of endothelial dysfunction, so the reduction of the DHE-derived signal by **1B** and, in particular, by **1C** (69% of control) suggests potential cytoprotective applications in this cellular context. It must be emphasized, however, that the redox measurements were performed exclusively in HMEC-1 endothelial cells, whereas the antiproliferative activity of the compounds was evaluated in cancer cell lines; the two sets of observations were therefore obtained in different cellular models and cannot be directly linked. In tumour cells, both an excess and a depletion of ROS can be antiproliferative, and accumulating evidence links flavonoid- and chromone-induced growth inhibition to redox modulation [15,21]; nevertheless, whether redox modulation contributes to the antiproliferative activity of **1B** and **1C** in cancer cells cannot be concluded from the present experiments and would require dedicated evaluation of the redox response in the relevant cancer cell lines. If such an association is confirmed in future studies, the **1B/1C** pair may constitute an attractive model system for mechanistic studies of chromone-redox relationships in cancer cells.

## 3. Materials and Methods

### 3.1. Materials

All reagents were purchased from Sigma-Aldrich (St. Louis, MO, USA) and Polish Chemical Reagents (Gliwice, Poland) and used without further purification. All synthesized compounds were purified by crystallization from methanol.

### 3.2. Synthesis

**Figure 6:**
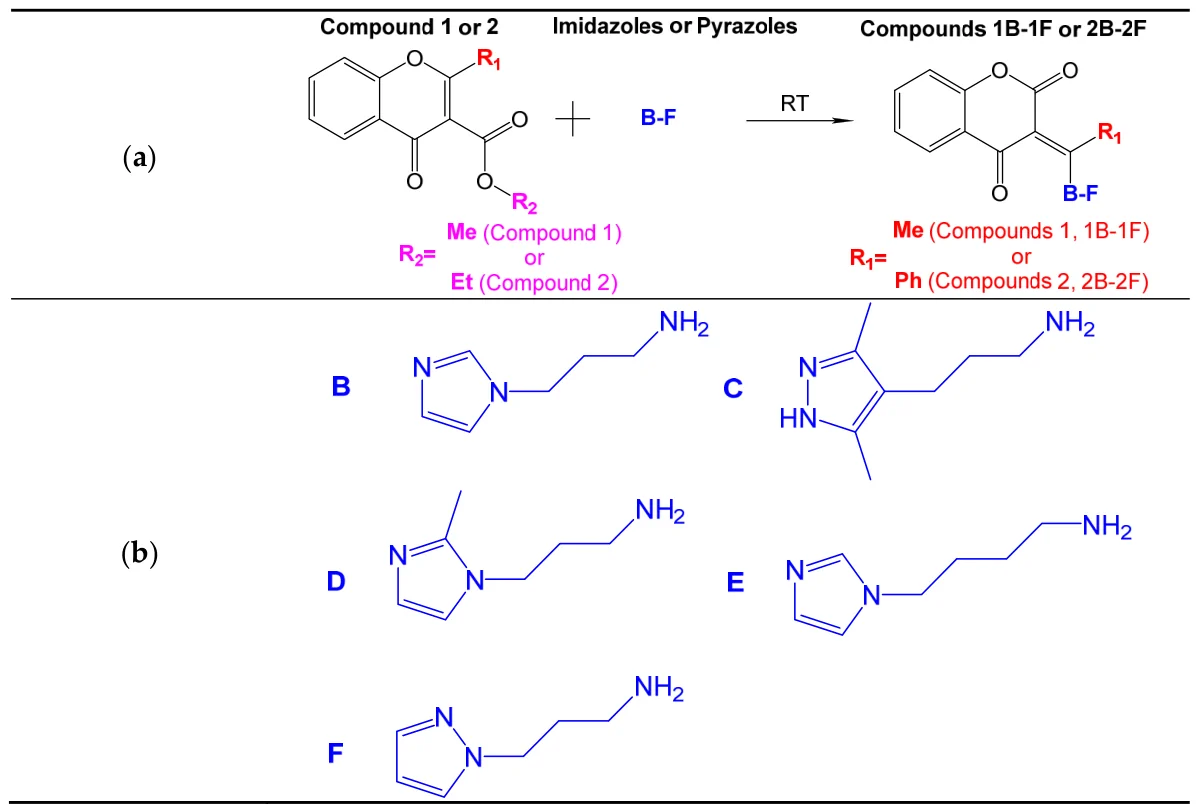
(**a**) Reaction scheme for the synthesis of chromone analogues; (**b**) structural formula of imidazole and pyrazoles.

The reactions were carried out as described previously [10]. The corresponding reaction scheme is shown in **Figure 6**.

#### 3.2.1. Compound **1B**

Yield: 66%, mp 126–127 °C. IR (KBr) υ(cm^−1^): 3411 (N-H), 3127, 3080 (C-H aromat.), 2918 (C-H aliph.), 1710 (C=O), 1611, 1575, 1487, 1469 (C=C), 1369, 1350 (C-H aliph.), 1322 (C-N), 1303, 1230, 1207, 1157, 1106, 1026 (C-O). ^1^*H*NMR (DMSO-d_6_) δ (ppm): 2.15 (2H, p, J_AB_ = 7.00 Hz NCH_2_C**H_2_**), 2.61 (3H, s, C**H_3_**), 3.56 (2H, t, J_AB_ = 7.20 Hz CNC**H_2_**), 4.09 (2H, t, J_AB_ = 7.00 Hz NC**H_2_**), 6.91–7.96 (7H, m, C-**H** aromat.), 13.77 (1H, s, N**H**). ^13^*C*NMR (DMSO-d_6_) δ (ppm): 18.13 (**C**1), 29.81 (**C**33), 41.08 (**C**32), 43.33 (**C**34), 96.20 (**C**3), 116.16 (**C**8), 119.18 (**C**10), 120.11 (**C**37), 123.59 (**C**6), 125.59 (**C**5), 128.57 (**C**36), 133.97 (**C**7), 137.23 (**C**35), 152.99. The numbering of carbon atoms in the ^13^*C*NMR spectrum description is consistent with **Figure 1a**. Anal. Calcd for C_17_H_17_N_3_O_3_ (M = 311.34 g/mol), %C 65.58%, %H 5.50%, %N 13.50%, %O 15.42%. Found: %C 65.53%, %H 5.69%, %N 13.90%, %O 15.88%.

#### 3.2.2. Compound **2B**

Yield: 65%, mp 133–135 °C IR (KBr) υ(cm^−1^): 3414 (N-H), 3106, 3065 (C-H aromat.), 2943, 2935 (C-H aliph.), 1716 (C=O), 1610, 1505, 1466 (C=C), 1345 (C-H aliph.), 1307 (C-N), 1226, 1207, 1145, 1108, 1079 (C-O). ^1^*H*NMR (DMSO-d_6_) δ (ppm): 2.02 (2H, p, J_AB_ = 6.70 Hz NCH_2_C**H_2_**), 3.12 (2H, t, J_AB_ = 7.10 Hz CNC**H_2_**), 3.95 (2H, t, J_AB_ = 7.00 Hz NC**H_2_**), 6.83–7.99 (12H, m, C-**H** aromat.), 13.61 (1H, s, N**H**). ^13^*C*NMR (DMSO-d_6_) δ (ppm): 30.31 (**C**33), 42.36 (**C**32), 43.04 (**C**34), 96.18 (**C**3), 116.35 (**C**8), 118.94 (**C**10), 120.18 (**C**39), 123.64 (**C**6, **C**5, **C**12), 125.60 (**C**14, **C**15), 126.07 (**C**11), 128.41 (**C**38), 128.45 (**C**1), 129.15 (**C**13), 133.33 (**C**7), 134.21 (**C**36), 137.04 (**C**9), 153.57 (**C**31), 174.79 (**C**2), 179.91 (**C**4). The numbering of carbon atoms in the ^13^*C*NMR spectrum description is consistent with **Figure 1b**. Anal. Calcd for C_22_H_19_N_3_O_3_ (M = 373.41 g/mol), %C 70.76%, %H 5.13%, %N 11.25%, %O 12.85%. Found: %C 70.73%, %H 5.00%, %N 11.40%, %O 12.87%.

#### 3.2.3. Compound **1C**

Yield: 41%, mp 164–165 °C. IR (KBr) υ(cm^−1^): 3208 (N-H), 3150, 3080 (C-H aromat.), 2920, 2858 (C-H aliph.), 1715 (C=O), 1561, 1451 (C=C), 1370 (C-H aliph.), 1328 (C-N), 1306, 1232, 1208, 1158, 1108, 1031 (C-O). ^1^*H*NMR (DMSO-d_6_) δ (ppm): 1.80 (2H, p, J_AB_ = 7.00 Hz NCH_2_C**H_2_**), 2.10 (6H, s, NNCC**H_3_**), 2.43 (2H, t, J_AB_ = 7.80 Hz CH_2_CH_2_C**H_2_**), 2.63 (3H, s, NHCC**H_3_**), 3.55 (2H, d, J_AB_ = 4.1 Hz NHC**H_2_**), 7.24–7.96 (4H, m, C-**H** aromat.), 11.91 (1H, s, CN**H**), 13.81 (1H, s, NN**H**). ^13^*C*NMR (DMSO-d_6_) δ (ppm): 18.9 (**C**1), 20.1 (**C**36A), 29.5 (**C**32), 41.1 (**C**33), 43.5 (**C**34), 96.5 (**C**3), 113.5 (**C**5), 116.8 (**C**8), 121.0 (**C**10), 124.1 (**C**7), 125.9 (**C**6), 134.5 (**C**9), 153.5 (**C**2), 162.5 (**C**31), 181.0 (**C**4). The numbering of carbon atoms in the ^13^*C*NMR spectrum description is consistent with **Figure 1c**. Anal. Calc for C_19_H_21_N_3_O_3_ (M = 339.40 g/mol), %C 67.24%, %H 6.24%, %N 12.38%, %O 14.14%. Found: %C 67.12%, %H 6.12%, %N 12.35%, %O 14.41%.

#### 3.2.4. Compound **1D**

Yield: 51%, mp 143–145 °C. IR (KBr) υ(cm^−1^): 3448 (N-H), 3131, 3080 (C-H aromat.), 2924 (C-H aliph.), 1700 (C=O), 1611, 1575, 1487, 1468 (C=C), 1375, 1349 (C-H aliph.), 1308 (C-N), 1275, 1231, 1209, 1142, 1088, 1030 (C-O). ^1^*H*NMR (DMSO-d_6_) δ (ppm): 2.09 (2H, p, J_AB_ = 7.50 Hz NCH_2_C**H_2_**), 2.29 (3H, s, NC(C**H_3_**)N), 2.63 (3H, s, C**H_3_**), 3.59 (2H, t, J_AB_ = 7.20 Hz CNC**H_2_**), 3.99 (2H, t, J_AB_ = 7.00 Hz NHC**H_2_**), 6.71–7.96 (6H, m, C-**H** aromat.). ^13^*C*NMR (DMSO-d_6_) δ (ppm): 12.45 (**C**39), 18.15 (**C**1), 29.43 (**C**34), 41.07 (**C**33), 42.44 (**C**35), 116.15 (**C**3), 119.27 (**C**8), 120.09 (**C**5), 123.59 (**C**36), 125.57 (**C**6), 126.14 (**C**10), 126.44 (**C**7), 133.96 (**C**38), 143.66 (**C**9), 152.97 (**C**2), 161.79 (**C**4), 176.38 (**C**31). The numbering of carbon atoms in the ^13^*C*NMR spectrum description is consistent with **Figure 1d**. Anal. Calcd for C_18_H_19_N_3_O_3_ (M = 325.37 g/mol), %C 66.45%, %H 5.89%, %N 12.91%, %O 14.75%. Found: %C 66.16%, %H 5.73%, %N 12.23%, %O 16.88%.

#### 3.2.5. Compound **1E**

Yield: 65%, mp 156–157 °C. IR (KBr) υ(cm^−1^): 3546 (N-H), 3103 (C-H aromat.), 2929 (C-H aliph.), 1687 (C=O), 1609, 1572, 1486, 1465 (C=C), 1375, 1343 (C-H aliph.), 1311 (C-N), 1283, 1231, 1163, 1141, 1079, 1029 (C-O). ^1^*H*NMR (DMSO-d_6_) δ (ppm): 1.60 (2H, p, *J*_AB_ = 7.20 Hz NCH_2_CH_2_C**H_2_**), 1.83 (2H, p, *J*_AB_ = 7.10 Hz NCH_2_C**H_2_**), 2.64 (3H, s, C**H_3_**), 3.61 (2H, t, *J*_AB_ = 7.00 Hz NHC**H_2_**), 4.04 (2H, t, *J*_AB_ = 7.20 Hz NC**H_2_**), 6.87–7.95 (7H, m, C-**H** aromat.), 13.74 (1H, s, N**H**).^13^*C*NMR (DMSO-d6) δ (ppm): 18.20 (**C**1), 25.41 (**C**33), 27.74 (**C**34), 43.16 (**C**32), 45.29 (**C**35), 96.05 (**C**3), 116.15 (**C**8), 119.16 (**C**10), 120.13 (**C**38), 123.57 (**C**6), 125.58 (**C**5), 128.40 (**C**37), 133.93 (**C**7), 137.20 (**C**36), 152.99 (**C**31), 161.81 (**C**2), 176.12 (**C**4). The numbering of carbon atoms in the ^13^*C*NMR spectrum description is consistent with **Figure 1e**. Anal. Calcd For C_18_H_19_N_3_O_3_ (M = 325.37 g/mol), %C 66.45%, %H 5.89%, %N 12.91%, %O 14.75%. Found: %C 66.09%, %H 6.16%, %N 13.00%, %O 15.75%.

#### 3.2.6. Compound **1F**

Yield: 45%, mp 106–108 °C. IR (KBr) υ(cm^−1^): 3432 (N-H), 3141, 3127 (C-H aromat.), 2942 (C-H aliph.), 1698 (C=O), 1614, 1575, 1486, 1468 (C=C), 1398, 1340 (C-H aliph.), 1301 (C-N), 1279, 1236, 1153, 1139, 1098, 1088 (C-O). ^1^*H*NMR (DMSO-d_6_) δ (ppm): 2.19 (2H, p, *J*_AB_ = 7.20 Hz NHCH_2_C**H_2_**), 2.61 (3H, s, C**H_3_**), 3.58 (2H, t, *J*_AB_ = 7.00 Hz NHC**H_2_**), 4.25 (2H, t, *J*_AB_ = 7.00 Hz NC**H_2_**), 6.25–7.97 (7H, m, C-**H** aromat.), 13.79 (1H, s, N**H**) ^13^*C*NMR (DMSO-d_6_) δ (ppm): 18.08 (**C**1), 29.35 (**C**33), 41.16 (**C**32), 48.21 (**C**34), 96.17 (**C**3), 105.15 (**C**38), 116.15 (**C**8), 120.12 (**C**5), 123.58 (**C**6), 125.59 (**C**10), 129.84 (C39), 133.94 (**C**7), 138.73 (**C**37), 152.98 (**C**9), 161.79 (**C**2), 176.32 (**C**4), 179.55 (**C**31). The numbering of carbon atoms in the ^13^*C*NMR spectrum description is consistent with **Figure 2**. Anal. Calcd For C_17_H_17_N_3_O_3_ (M = 311.34 g/mol), %C 65.58%, %H 5.50%, %N 13.50%, %O 15.42%. Found: %C 65.17%, %H 5.50%, %N 13.47%, %O 15.86%.

### 3.3. Single-Crystal X-Ray Measurements

The compounds were subjected to single crystal X-ray analysis using a micro-focus source sealed X-ray tube; the scan was performed using CuKα on an XtaLAB Synergy S, Duaflex, HyPix diffractometer with ω scan at low temperature (100–102 K). The X-ray data were corrected using CrysAlisPro [11]. All structures were solved using SHELXT [12], refined with SHELXL-2014/7 [13] using WinGX software v2021.3 [21] and Olex2 software ver. 1.5 [14]. Other calculations and operations were performed using Platon [22] and Mercury [23]. All non-hydrogen atoms were refined anisotropically. All hydrogen atoms were located according to the rigid body model. The crystal quality of compound 1D was poor, as reflected in the R and wR values. Other crystallographic data for the structures reported in this paper may be obtained free of charge on application to CCDC, 12 Union Road, Cambridge CB2, 1EZ, UK [fax: (44) 1223-336-033; email: deposit@ccdc.cam.ac.uk] quoting the depository numbers: 2590051, 2590048, 2590050, 2590054, 2590052, 2590053.

### 3.4. Hirshfeld Surface Analysis

To examine the intermolecular interactions in the crystal structures of the synthesized chromone analogues, Hirshfeld surface analysis was performed using CrystalExplorer21 [24]. The surfaces were mapped over d_norm_ (range −1.0 to 2.0 Å), and the associated two-dimensional fingerprint plots were analysed to evaluate the contributions of different intermolecular contacts. The d_i_ and d_e_ values correspond to the distances from the Hirshfeld surface to the nearest atoms inside and outside the surface, respectively. The red, white, and blue regions on the Hirshfeld surface denote contacts that are, respectively, shorter than, equal to, and longer than the sum of the corresponding van der Waals radii.

### 3.5. Isothermal Titration Calorimetry (ITC)

Interactions of the selected chromone analogues (Chromono-3-carboxylic acid esters and their pyrazole/imidazole derivatives) with β-cyclodextrin were studied with ITC titrations, using VP-ITC microcalorimeter (MicroCal, Northamton, MA, USA). Since very low aqueous solubility of the studied chromone analogues, all titrations were performed in DMSO solvent. In a typical titration, a 0.4 mM solution of chromone analogues in DMSO in the calorimetric cell (working volume 1427 μL) was titrated from the syringe with 50 aliquots of 10 mM β-cyclodextrin solution in the same solvent (5 μL volume each). The duration of a single injection was 10 s, and the time between successive injections was 500 s. Measurements were carried out at a constant temperature (25 °C) at the stirring rate of 416 rpm.

During the titration, the molar ratio of β-CD to chromone derivative increased from 0.02/1 to 4.7/1. For background correction, pure DMSO (in the cell) was titrated with 10 mM β-cyclodextrin solution (from the syringe); the value was then subtracted from the obtained heat effects of the titration of chromone derivatives with β-cyclodextrin. The obtained enthalpograms of direct interactions between chromone derivatives and β-cyclodextrin in DMSO were analysed by nonlinear multiparameter regression using MicroCal Origin software, ver. 7.0.

All synthesized compounds were subjected to ITC titrations. However, apparent complex stoichiometry and binding parameters could only be obtained for Compounds **1** and **1A** by fitting the data to the one set of binding sites model. Other models, including two sets of binding sites and sequential binding do not describe the obtained experimental data. Since Compound **1** (as a substrate) contains an ester bond, whereas the other derivatives obtained as condensation reaction products lack this bond, we decided to also test Compound **1A (Figure S15)**. The molecule of Compound **1A** is more sterically developed as it consists of a 1,2,3-triazole ring fused with a methyl ester of 3-chromonecarboxylic acid. The interaction of Compound **1A** with β-CD in solution has not been previously investigated with the ITC technique. The use of Compound **1A** allowed us to compare the impact of expanding the substrate—Compound **1**—on the host–guest interaction with β-CD.

### 3.6. Molecular Docking

Molecular docking was performed to understand the host–guest interactions between cyclodextrin and the newly synthesized compounds; Compound **1** and Compounds **1A**–**1F**, Compound **2** and Compounds **2A**–**2F**. Since Compound **1** contains an ester group, unlike the other derivatives obtained through the condensation reaction, Compound **1A** was additionally selected for investigation. This is the first molecular docking analysis of the interaction of Compound **1A** with β-CD. The inclusion of Compound **1A** in the study enabled us to assess how structural expansion of the chromone derivative affects its host–guest interactions with β-CD.

Cyclodextrin complexation was employed because it temporarily accommodates hydrophobic portions of drug molecules inside a water-compatible carrier, improving properties such as solubility, dissolution, stability, and sometimes bioavailability. To predict the preferred orientation and stability of each cyclodextrin-ligand complex, docking is carried out using AutoDock Vina Version 1.1.2 with a blind-docking approach. All ligands and *β*-cyclodextrin were optimized and then prepared by removing nonpolar hydrogens and assigning Gasteiger charges, and converted to PDBQT format. A 30 × 30 × 30 Å^3^ grid was applied to cover the entire cyclodextrin cavity, and an exhaustiveness level of 6 was used. The top three binding modes for each compound were visualized and analyzed in PyMOL Version 3.1 to assess hydrophobic insertion, hydrogen bonding, and cavity fit.

The crystallographic structures provided experimentally determined starting geometries for the ligands; however, the resulting docking poses represent computational predictions of possible host–guest arrangements rather than experimentally determined structures of the β-CD inclusion complexes. Furthermore, the docking calculations considered one ligand and one β-CD molecule at a time. Consequently, the docking results were interpreted as providing qualitative information about possible binding orientations and intermolecular interactions, rather than direct evidence of the stoichiometry or complete structure of higher-order complexes.

### 3.7. Biological Material, Culture and Passage of Cells

To evaluate the cytotoxicity of the synthesized chromone derivatives, experiments were conducted using a panel of human tumor cell lines alongside a non-cancerous reference model. The cancer models comprised three breast adenocarcinoma lines, *viz.* estrogen and progesterone receptor-positive MCF-7 (ATCC^®^ HTB-22™) and receptor-negative MDA-MB-231 (ATCC^®^ HTB-26™) and HCC38 (ATCC^®^ CRL-2314™), and two endometrial carcinoma lines, *viz.* Ishikawa (99040201; Sigma–Aldrich, St. Louis, MO, USA) and Hec-1-A (ATCC^®^ HTB-112™). Human microvascular endothelial cells, HMEC-1 (ATCC, Manassas, VA, USA), served as the normal cell control. Each cell line was grown in its respective optimized medium. HMEC-1 cells were maintained in MCDB 131 formulation (Corning Life Sciences, Corning, NY, USA) supplemented with 10% fetal bovine serum, 10 U/mL penicillin, and 0.5 mg/mL streptomycin (Gibco, Grand Island, NY, USA), alongside 10 mM L-glutamine, 1 μg/mL hydrocortisone, and 10 ng/mL epidermal growth factor (EGF) (Millipore, Burlington, MA, USA). The MCF-7, MDA-MB-231, Ishikawa, and Hec-1-A lines were cultured in Dulbecco’s Modified Eagle Medium (DMEM; Lonza, Visp, Switzerland), whereas HCC38 cells were kept in RPMI medium (Thermo Fisher Scientific, Waltham, MA, USA). Both DMEM and RPMI media were supplemented with 10% FBS and the penicillin-streptomycin antibiotic mixture (10 U/mL and 0.5 mg/mL, respectively). All cultures were incubated under standard environmental parameters of 37 °C, 5% CO_2_ atmosphere, and 100% relative humidity. The exponential growth phase was maintained by subculturing two to three times per week using 0.25% trypsin-EDTA (Gibco). Briefly, the culture supernatant was discarded, and the cell monolayer was washed with 0.9% NaCl solution. Enzymatic detachment was initiated by adding 300–500 μL of trypsin-EDTA, scaled to the vessel surface area, and incubated for 3–5 min while monitoring detachment under a microscope. Once detached, the trypsin solution was gently aspirated, and the cell pellet was resuspended in fresh, complete growth medium. The homogeneous cell suspension was subsequently seeded into sterile culture vessels at optimized densities for subsequent assays.

### 3.8. Determination of Cell Viability by Metabolic Microplate Spectrophotometric Assay with MTT

To evaluate the in vitro antiproliferative activity of the synthesized compounds, cell viability was measured using the colorimetric MTT (3-(4,5-dimethyl-2-thiazolyl)-2,5-diphenyl-2*H*-tetrazolium bromide) metabolic assay (Sigma-Aldrich, St. Louis, MO, USA). Cells from each line were harvested and seeded into sterile, clear 96-well microplates at a density of 8 × 10^3^ cells/mL. Following a 24 h incubation period, the cells were exposed to varying concentrations of the test compounds (0–120 µmol/L) in designated wells and incubated under CO_2_ conditions for 24 h, after which the MTT assay was performed directly on the compound-treated cells; the IC_50_ values reported in this study therefore reflect a 24 h exposure to the test compounds. Following this, 50 µL of the tetrazolium salt solution was introduced to each well to achieve a final concentration of 0.05 mg/mL, and the microplates were returned to the CO_2_ incubator for three to four hours, depending on the specific cell line requirements.

Next, 100 µL of dimethyl sulfoxide (DMSO; Sigma-Aldrich, St. Louis, MO, USA) was added per well to solubilize the insoluble purple formazan crystals generated by viable cells. Following brief mechanical shaking, optical density was measured at a primary wavelength of λ = 580 nm and a reference wavelength of λ = 720 nm using a BioTek PowerWave HT microplate reader (BioTek Instruments, Winooski, VT, USA). The percentage of viable cells was determined relative to untreated control wells, which were defined as representing 100% cell viability. From these data, IC_50_ values representing the compound concentration required to reduce cell viability by 50% were calculated. IC_50_ values were reported as precise estimates only when the fitted concentration–response curve reliably crossed the 50% viability level; when the curve did not cross the 50% viability level within the tested concentration range (0–120 µM), the IC_50_ value is reported as >120 µM (Table 4). All IC_50_ values reported as precise estimates in Table 4, including those close to the upper limit of the tested range, meet this criterion. Selectivity indices (SI) were calculated from the mean IC_50_ values as SI = IC_50_ (HMEC-1)/IC_50_(cancer cell line), where an SI > 1 indicates a preferential cytotoxic effect of the compound on a given cancer cell line over the non-cancerous HMEC-1 reference line. As the IC_50_ values were determined within a limited concentration range (up to 120 µM), an exact SI could not be obtained when the IC_50_ of a cancer cell line and/or of HMEC-1 exceeded 120 µM; in such cases, boundary estimates (SI > 120/IC_50_(cancer cell line) or SI < IC_50_(HMEC-1)/120) are reported, and when both IC_50_ values exceeded the tested range, the SI was not calculated (n.c.).

### 3.9. Determination of Antioxidant Potential Using Fluorescent Probes DHE and H_2_DCFDA

In order to assess the antioxidant potential of the analyzed derivatives, two fluorescent probes were used: dihydroethidium (DHE) displays blue fluorescence in the cytosol, transitioning to bright red upon oxidation to ethidium and subsequent DNA intercalation. This shift facilitates the use of DHE as a sensitive probe for cellular reactive oxygen species (ROS). The reaction of DHE with superoxide generates 2-hydroxyethidium (2-OH-E+); however, DHE can also be oxidized to several other fluorescent products (e.g., ethidium and its dimers), and the total DHE-derived signal should therefore be interpreted as an overall indicator of the oxidation processes reported by this probe rather than as a specific quantitative measure of superoxide [17,18]. 2′,7′-dichlorodihydrofluorescein diacetate (H_2_DCFDA, Sigma-Aldrich; St. Louis, MO, USA) is a widely used redox-sensitive probe; however, its oxidation is not selective for hydrogen peroxide, and the DCF-derived signal should be interpreted as an overall redox-sensitive readout rather than a selective measure of cellular H_2_O_2_ [17,19]. In the form of a reduced fluorescent probe, it freely diffuses through the cell membrane. Inside the cell, it is hydrolyzed (deacetylated) to non-fluorescent H_2_DCF under the influence of esterases. H_2_DCF is then oxidized in the presence of the generated ROS, forming 2’,7’-dichlorofluorescein (DCF)—a product with strong fluorescence. DCF accumulates in the cytosol, showing characteristic excitation and emission maxima at λ_ex_ = 498 nm and λ_em_ = 522 nm. The fluorescence intensity of DCF is proportional to the concentration of ROS in the cell.

In order to assess the antioxidant potential of the analyzed derivatives, two fluorescent probes were used: dihydroethidium (DHE) and 2′,7′-dichlorodihydrofluorescein diacetate (H2DCFDA, Sigma-Aldrich; St. Louis, MO, USA). The cells were seeded in black 96-well fluorometric microplates at a density of 10 × 10^3^ cells per well 24 h before the treatment. After this time, the cells were preincubated for 24 h with the test compounds (1B or 1C) at a concentration of 50 µmol/L; cells incubated with the vehicle alone served as the non-treated control. The medium was then removed, the cell monolayer was washed twice with HBSS (140 mM NaCl, 5 mM KCl, 0.8 mM MgCl_2_, 1.8 mM CaCl_2_, 1 mM Na_2_HPO_4_, 10 mM HEPES and 1% glucose), and the cells were challenged with 50 µmol/L H_2_O_2_ for 30 min to induce oxidative stress; H_2_O_2_ was not added to the non-treated control. Following the challenge, the cells were washed twice with HBSS, and 50 µL of DHE/H_2_DCFDA solution in HBSS was added to each well (final probe concentration: 5 µmol/L). The cells were incubated with the probes for 30 min in the dark at 37 °C. Fluorescence was then measured at λ_ex_ = 498 nm/λ_em_ = 582 nm (DHE) or λ_ex_ = 498 nm/λ_em_ = 522 nm (H_2_DCFDA) using a Perkin-Elmer LS-5B spectrofluorometer (Massachusetts, USA). The fluorescence intensity of the control cells was arbitrarily set at 100%, and the results were expressed as percentages of the control (sample fluorescence was calculated as Δf relative to the control).

### 3.10. Statistical Analysis

All experiments were performed in three independent replicates (n = 3). Data from the MTT assay are presented as the mean ± standard error of the mean (SEM), and data from the fluorescence measurements as the mean ± standard deviation (SD). IC_50_ values were derived from the concentration–response curves by nonlinear regression and are reported with the standard error of the fit; when the curve did not cross 50% viability within the tested concentration range, the value is reported as >120 µM. Fluorescence data were analysed by one-way analysis of variance (ANOVA) followed by Dunnett’s post hoc test for comparisons with the untreated control, with *p* < 0.05 considered statistically significant.

## 4. Conclusions

This article continues the research on chromone derivatives, complementing and substantially expanding upon previously presented results (**Table S7**) [10]. All six new compounds were unambiguously characterized by single-crystal X-ray diffraction. The molecules consist of a planar benzopyran-4-one core carrying an imidazole- or pyrazole-aminopropyl substituent at C-3, and their conformations are governed by N–H···O and C–H···O hydrogen bonds, with methanol or water molecules of crystallization incorporated in the lattices of **1B**, **1E** and **1F**. Hirshfeld surface analysis identified H···H (44.6–49.6%), O···H and C···H contacts as the dominant contributions to the crystal packing.

Among the currently investigated chromone-3-carboxylic acid esters and their pyrazole/imidazole derivatives, the apparent parameters of their complexation by β-cyclodextrin in DMSO at 25 °C were determined for only two derivatives—Compound **1** and Compound **1A**. Isothermal titration calorimetry revealed that Compound 1 and 1A form inclusion complexes with β-cyclodextrin, where the calculated stoichiometric parameters N ≈ 2.6, suggests that additional β-CD macrocycle interacts with the complexed with β-CD guests—Compounds **1** and **1A**—in DMSO. β-Cyclodextrin can spontaneously self-assemble to form CD aggregates, especially after inclusion complex is formed, in polar solvents, like water [25] or DMSO [26]. Both binding of Compounds **1** and **1A** with β-cyclodextrin and the proposed interactions of the formed inclusion complex with additional β-CD macrocycle is a weak endothermic, entropy-driven process (ΔS > 0) in DMSO solvent. Endothermic enthalpy of β-CD binding with Compounds **1** and **1A** indicates that partial desolvation and solvent release, rather than direct host–guest enthalpic interactions, drive the complexation; the favourable blind-docking scores (−5.3 to −6.6 kcal/mol) corroborate these findings. An aqueous environment is frequently chosen as the medium for studying the complexation processes of β-CD by water-soluble bioactive ligands, such as flurbiprofen [27]. ITC calorimetric studies of the interactions of β-cyclodextrin with molecules of poorly water-soluble ligands are often conducted in a DMSO environment (**Table S7**) [28,29,30], whose highly polar molecules interact with the ligand molecules to enable their dissolution. This solvent was used, among others, to evaluate the interactions of dimers of permethylated β-cyclodextrin with tetracene [31] and anionic β-cyclodextrin derivatives with cationic ligands [32]. To date, exothermic binding of ligands by (unsubstituted) β-cyclodextrin has been observed in DMSO at 25 °C, forming complexes with a predominant stoichiometry close to 1:1 (**Table S7**), particularly for chromanone analogues without an ester bond (respectively **3**, **3A** and **5** in **Table S7**), flavanones (**Table S7**), and ezetimibe (**Table S7**), which was accompanied by an increase in complexation entropy.

The obtained results indicate that the ester bond and the substituents introduced into the structure of the chromone derivatives influence the complexation process of these ligands within the β-cyclodextrin cavity, which differs from previously studied ligands (**Table S7**). The binding of Compound **1** and Compound **1A** derivatives with β-CD in DMSO at 25 °C is an endothermic process and occurs thermodynamically spontaneously due to an increase in the degree of disorder of the reactants, which compensates for the endothermic enthalpy of complexation. The endothermic nature of the binding of Compound **1** and Compound **1A** with β-CD indirectly indicates their stronger solvation by DMSO compared to the chromanone analogues with β–CD studied previously (**Table S7**), for which exothermic complexation was observed. The complexed with β-cyclodextrin molecules of Compound **1** and Compound **1A** interact further with proposed additional molecules of β-cyclodextrin stabilizing the formed apparent higher-order association, as evidenced by a higher interaction constant, higher interaction entropy, and lower free energy of proposed interaction for Compound **1** and Compound **1A** with β–CD than in the case of chromanone analogues without an ester bond (respectively **3**, **3A** and **5** in **Table S7**) with β–CD and ezetimibe with β–CD in DMSO at 25 °C (**Table S7**). The biological evaluation identified Compounds **1B** and **1C**, bearing the 3-(1H-imidazol-1-yl)propan-1-amine and 3-(3,5-dimethyl-4*H*-pyrazol-4-yl)propan-1-amine side chains, as the most active derivatives of the series. In the MTT assay they inhibited the proliferation of both the oestrogen-receptor-positive MCF-7 and the triple-negative MDA-MB-231 breast cancer cells, with IC_50_ values of 81.2–82.5 µM and 87.0–91.5 µM, respectively, whereas the remaining analogues (**1D–1F**, **2B**) were essentially inactive, with IC50 values above 120 µM in the majority of the tested cell lines. The selectivity of Compounds **1B** and **1C** towards cancer cells over the non-cancerous HMEC-1 line was, however, limited (SI = 1.01–1.47 for **1B** and 0.87–1.24 for **1C;** **Table 4**). The activity of **1B** and **1C** falls within the concentration range reported for chromone- and flavonoid-based cytotoxic agents of comparable structural complexity—the parent flavonol quercetin inhibits MCF-7 proliferation with IC_50_ values of 23–73 µM [16]—and was determined after a 24 h exposure; the reported values therefore characterize the antiproliferative activity under this specific protocol, and direct comparison with literature data obtained under longer exposure times (48–72 h) should be made with caution. Nevertheless, in view of the moderate potency and the limited selectivity indices, Compounds **1B** and **1C** should be regarded as starting points for further structural optimization rather than as promising anticancer drug candidates. The structure–activity relationship is unambiguous: methylation at the 2-position of the imidazole ring (**1D**), elongation of the spacer to a butyl chain (**1E**), replacement of imidazole by pyrazole (**1F**) and enlargement of the ester group (**2B**) all abolish the activity. Importantly, the less active analogues were essentially non-toxic towards the non-cancerous HMEC-1 line, and the two active compounds displayed only a limited preference for cancer cells over the endothelial reference (SI ≤ 1.47).

A noteworthy observation is that the same two compounds modulate the redox state of HMEC-1 cells: they lowered the DHE-derived signal to 85% and 69% of the control (for **1B** and **1C**, respectively) without altering the H_2_DCFDA readout and without any sign of pro-oxidant behaviour. Because the selectivity of the applied probes is limited, this observation should be interpreted as a modulation of the redox processes reported by the DHE probe rather than as evidence for a specific effect on superoxide homeostasis. Since the redox experiments were conducted in non-cancerous endothelial cells, this finding is potentially relevant to cytoprotection in such cells, where superoxide is a key mediator of endothelial dysfunction; it should, however, be clearly separated from the antiproliferative activity of **1B** and **1C** observed in cancer cell lines. Whether redox modulation contributes to the antiproliferative activity of these compounds in cancer cells remains an open question and requires dedicated studies in the relevant cellular models [32].

The moderate antiproliferative potency of **1B** and **1C** nevertheless indicates that the substitution pattern explored here should be treated as a starting point rather than an optimized pharmacophore, and further synthetic efforts are required to fully develop the cytotoxic potential of this scaffold. The most promising directions include increasing the hydrophobic and aromatic content at the C-3 substituent, systematic variation in the linker length and imidazole/pyrazole N-substitution, and introduction of electron-withdrawing groups on the chromone benzene ring, guided by the clear SAR outlined above. The favourable inclusion behaviour of the chromone esters in β-cyclodextrin suggests that cyclodextrin complexation may additionally serve as a solubility-enabling formulation for the poorly water-soluble analogues, and the **1B/1C** pair may constitute an attractive model system for mechanistic studies linking chromone chemistry with redox signalling, provided that the redox response is first evaluated in the relevant cancer cell lines.

## Figures and Tables

**Table 2:** Thermodynamic parameters of the formation of supramolecular complexes of chromone derivatives (Compound **1** and Compound **1A**) with β-CD in DMSO at 25 °C determined by ITC.

Chromone Derivative	n	logK	∆H [cal mol^−1^]	∆S [cal K^−1^ mol^−1^]	∆G [kcal mol^−1^]
**Compound 1**	2.6 ± 0.1	4.86 ± 0.21	93 ± 4	22.6 ± 0.9	–6.4 ± 0.3
**Compound 1A**	2.6 ± 0.2	4.08 ± 0.28	28 ± 2	18.8 ± 1.1	–5.6 ± 0.3

**Table 3:** Binding affinities (E_b_, kcal/mol) of the studied chromone derivatives towards β-CD.

Modes (kcal/mol)			
Compound	1st	2nd	3rd
Compound **1**	−5.5	−5.4	−5.3
Compound **2**	−5.6	−5.4	−5.4
Compound **1A**	−6.4	−6.3	−6.3
Compound **2A**	−6.2	−6.1	−6.1
Compound **1B**	−6	−6	−6
Compound **2B**	−6	−5.9	−5.9
Compound **1C**	−6.3	−6.1	−6.1
Compound **2C**	−6.6	−6.5	−6.3
Compound **1D**	−5.9	−5.9	−5.7
Compound **2D**	−5.9	−5.9	−5.8
Compound **1E**	−5.7	−5.7	−5.6
Compound **2E**	−6	−6	−5.8
Compound **1F**	−5.8	−5.7	−5.7

**Table 4:** *In vitro* cytotoxicity of six chromone analogues (Compounds **1B**, **1C**, **1D**, **1E**, **1F** and **2B**) in various human cancer cells and the immortalized human microvascular endothelial cell line HMEC-1 as a model of normal cells. The cells were incubated with the compounds for 24 h with a wide range of test compound concentrations (0–120 µM). Results are expressed as the mean ± SEM of three repeated experiments. IC_50_ values (μM) were calculated at the concentration of a tested compound required to reduce the fraction of surviving cells to 50% of that observed in comparison to the control probe (non-treated cell). Selectivity index (SI), defined as the ratio of the mean IC_50_ value determined in the non-cancerous HMEC-1 line to the mean IC_50_ value determined in a given cancer cell line, is shown for each compound in the row denoted SI. Values marked with an inequality sign (< or >) are boundary estimates arising when one of the IC_50_ values exceeds the maximum tested concentration (120 µM), and are rounded to two decimal places; n.c.—not calculable (both IC_50_ values above the tested range);—not applicable (HMEC-1 is the reference line).

Cytotoxic Effect, IC_50_ (μM) and Selectivity Index (SI)						
	MCF7	MDA-MB-231	HCC38	Ishikawa	Hec-1A	HMEC-1
Compound **1B**	81.2 ± 1.6	91.5 ± 1.9	118.2 ± 2.2	>120	114.8 ± 4.9	119.1 ± 1.4
***SI (1B)***	1.47	1.30	1.01	<0.99	1.04	—
Compound **1C**	82.5 ± 2.1	87 ± 3.2	116.9 ± 2.8	>120	117.9 ± 6.5	102.5 ± 2.1
***SI (1C)***	1.24	1.18	0.88	<0.85	0.87	—
Compound **1D**	>120	>120	>120	>120	>120	>120
*SI (1D)*	n.c.	n.c.	n.c.	n.c.	n.c.	—
Compound **1E**	113.4 ± 2.7	>120	119.8 ± 4.6	>120	>120	>120
***SI (1E)***	>1.06	n.c.	>1.00	n.c.	n.c.	—
Compound **1F**	114.1 ± 5.9	>120	>120	119.6 ± 5.1	>120	>120
***SI (1F)***	>1.05	n.c.	n.c.	>1.00	n.c.	—
Compound **2B**	>120	>120	>120	118.2 ± 2.7	>120	>120
***SI (2B)***	n.c.	n.c.	n.c.	>1.02	n.c.	—

## Data Availability

The original contributions presented in this study are included in the article/Supplementary Materials. Further inquiries can be directed to the corresponding author.

## Supplementary Materials

The following supporting information can be downloaded at https://www.mdpi.com/article/10.3390/molecules31213990/s1, Tables S1–S7: Hydrogen bond geometry; Figures S1–S15: Structural motifs and crystal packing (Refs. [10,28,29,30]).

## References

[1] Benny A.T., Arikkatt S.D., Vazhappilly C.G., Kannadasan S., Thomas R., Leelabaiamma M.S.N., Radhakrishnan E.K., Shanmugam P., Chromone A. (2022). Privileged Scaffold in Drug Discovery: Developments in the Synthesis and Bioactivity. Mini-Rev. Med. Chem..

[2] Mohsin N.u.A., Irfan M., Hassan S.u., Saleem U. (2020). Current Strategies in Development of New Chromone Derivatives with Diversified Pharmacological Activities: A Review. Pharm. Chem. J..

[3] Keri R.S., Budagumpi S., Pai R.K., Balakrishna R.G. (2014). Chromones as a Privileged Scaffold in Drug Discovery: A Review. Eur. J. Med. Chem..

[4] Reis J., Gaspar A., Milhazes N., Borges F. (2017). Chromone as a Privileged Scaffold in Drug Discovery: Recent Advances. J. Med. Chem..

[5] dos Santos Silva Araújo L., Lazzara G., Chiappisi L. (2021). Cyclodextrin/Surfactant Inclusion Complexes: An Integrated View of Their Thermodynamic and Structural Properties. Adv. Colloid Interface Sci..

[6] Loftsson T., Brewster M.E. (2010). Pharmaceutical Applications of Cyclodextrins: Basic Science and Product Development. J. Pharm. Pharmacol..

[7] Cid-Samamed A., Rakmai J., Mejuto J.C., Simal-Gandara J., Astray G. (2022). Cyclodextrins Inclusion Complex: Preparation Methods, Analytical Techniques and Food Industry Applications. Food Chem..

[8] Li T., Guo R., Zong Q., Ling G. (2022). Application of Molecular Docking in Elaborating Molecular Mechanisms and Interactions of Supramolecular Cyclodextrin. Carbohydr. Polym..

[9] Cai W., Xia B., Shao X., Guo Q., Maigret B., Pan Z. (2001). Molecular Docking of α-Cyclodextrin Inclusion Complexes by Genetic Algorithm and Empirical Binding Free Energy Function. Chem. Phys. Lett..

[10] Czapnik P., Adamus-Grabicka A., Budzisz E., Domagała M., Małecka M. (2026). Noncovalent Interactions and Crystal Packing Chromono-3-Carboxylic Acid Esters and Their Triazole Derivatives: A Combined Experimental and Theoretical Study. Cryst. Growth Des..

[11] Agilent Technologies Ltd. (2014). CrysAlisPro, Agilent 2014.

[12] Sheldrick G.M. (2015). SHELXT - Integrated Space-Group and Crystal-Structure Determination. Acta Crystallogr. A Found. Adv..

[13] Sheldrick G.M. (2015). Crystal Structure Refinement with SHELXL. Acta Crystallogr. C Struct. Chem..

[14] Dolomanov O.V., Bourhis L.J., Gildea R.J., Howard J.A.K., Puschmann H. (2009). OLEX2: A Complete Structure Solution, Refinement and Analysis Program. J. Appl. Crystallogr..

[15] Wu Q., Needs P.W., Lu Y., Kroon P.A., Ren D., Yang X. (2018). Different Antitumor Effects of Quercetin, Quercetin-3′-Sulfate and Quercetin-3-Glucuronide in Human Breast Cancer MCF-7 Cells. Food Funct..

[16] Hassanin N.M., Ali T.E., Assiri M.A., Abdel-Kariem S.M. (2024). Novel Carbazolyl–Thiazolyl–Chromone and Carbazolyl–Thiazolyl–Pyrazole Hybrids: Synthesis, Cytotoxicity Evaluation and Molecular Docking Studies. RSC Adv..

[17] Kalyanaraman B., Hardy M., Podsiadly R., Cheng G., Zielonka J. (2017). Recent Developments in Detection of Superoxide Radical Anion and Hydrogen Peroxide: Opportunities, Challenges, and Implications in Redox Signaling. Arch. Biochem. Biophys..

[18] Zhao H., Joseph J., Fales H.M., Sokoloski E.A., Levine R.L., Vasquez-Vivar J., Kalyanaraman B. (2005). Detection and Characterization of the Product of Hydroethidine and Intracellular Superoxide by HPLC and Limitations of Fluorescence. Proc. Natl. Acad. Sci. USA.

[19] Kalyanaraman B., Darley-Usmar V., Davies K.J.A., Dennery P.A., Forman H.J., Grisham M.B., Mann G.E., Moore K., Roberts L.J., Ischiropoulos H. (2012). Measuring Reactive Oxygen and Nitrogen Species with Fluorescent Probes: Challenges and Limitations. Free Radic. Biol. Med..

[20] Szalóki D.V., Szabó M., Bonczidai-Kelemen D., Udvardy A., May N.V., Kovács K., Virág L., Fábián I., Lihi N. (2025). Chromone-Based Antioxidant Copper(II) Complex with High Superoxide Dismutase Activity. Inorg. Chem..

[21] Farrugia L.J. (2012). WinGX and ORTEP for Windows: An Update. J. Appl. Crystallogr..

[22] Spek A.L. (2009). Structure Validation in Chemical Crystallography. Acta Crystallogr. D Biol. Crystallogr..

[23] Macrae C.F., Sovago I., Cottrell S.J., Galek P.T.A., McCabe P., Pidcock E., Platings M., Shields G.P., Stevens J.S., Towler M. (2020). Mercury 4.0: From Visualization to Analysis, Design and Prediction. J. Appl. Crystallogr..

[24] Spackman P.R., Turner M.J., McKinnon J.J., Wolff S.K., Grimwood D.J., Jayatilaka D., Spackman M.A. (2021). CrystalExplorer: A Program for Hirshfeld Surface Analysis, Visualization and Quantitative Analysis of Molecular Crystals. J. Appl. Crystallogr..

[25] Ryzhakov A., Do Thi T., Stappaerts J., Bertoletti L., Kimpe K., Sá Couto A.R., Saokham P., Van den Mooter G., Augustijns P., Somsen G.W. (2016). Self-Assembly of Cyclodextrins and Their Complexes in Aqueous Solutions. J. Pharm. Sci..

[26] Zhang H., Tan T., Feng W., van der Spoel D. (2012). Molecular Recognition in Different Environments: β-Cyclodextrin Dimer Formation in Organic Solvents. J. Phys. Chem. B.

[27] Betlejewska-Kielak K., Bednarek E., Budzianowski A., Michalska K., Maurin J.K. (2023). Comprehensive Characterisation of the Flurbiprofen/β-Cyclodextrin Inclusion Complex Using X-Ray Techniques and NMR Spectroscopy. J. Mol. Struct..

[28] Biernacka M., Ilyich T., Zavodnik I., Pałecz B., Stepniak A. (2022). Studies of the Formation and Stability of Ezetimibe-cyclodextrin Inclusion Complexes. Int. J. Mol. Sci..

[29] Adamus-Grabicka A.A., Hikisz P., Stepniak A., Malecka M., Paneth P., Sikora J., Budzisz E. (2024). Molecular Pro-Apoptotic Activities of Flavanone Derivatives in Cyclodextrin Complexes: New Implications for Anticancer Therapy. Int. J. Mol. Sci..

[30] Stepniak A., Biernacka M., Malecka M., Palecz B. (2024). Host-Guest Complexes of Flavanone and 4′-Chloroflavanone with Naturals and Modified Cyclodextrin: A Calorimetric and Spectroscopy Investigations. Molecules.

[31] Lebedinskiy K., Lobaz V., Jindřich J. (2022). Preparation of β-Cyclodextrin-Based Dimers with Selectively Methylated Rims and Their Use for Solubilization of Tetracene. Beilstein J. Org. Chem..

[32] Fotiadou K., Thanassoulas A., Nounesis G., Yannakopoulou K. (2011). Cooperative Heterodimer Formation between Per-Guadinylated and Carboxylated or Phosphorylated Cyclodextrins in DMSO and DMSO–Water Studied by NMR Spectroscopy and Microcalorimetry. Supramol. Chem..

