# Supplementary material for: New Chromone Analogues as Potential Bioactive Compounds: Cyclodextrin Complexation and Molecular Docking
Source: Molecules. 2026 Nov 5;31(21):3990. doi: 10.3390/molecules31213990 (PMC13764725; doi:10.3390/molecules31213990)
Supplement: Supplementary file 1 [file molecules-31-03990-s001.zip › molecules-4629400-supplementary.pdf]

Supplementary materials

New Chromone Analogues as Potential Bioactive Compounds: Cyclodextrin Complexation and Molecular Docking

Patryk Czapnik[1,2], Magdalena Małecka[1], Pawel Hikisz[3], Adam Buczkowski[1], Artur Stępniak[1], Khalida Khalil[1,2], Rehana Bano[4], Elzbieta Budzisz[5], Angelika A. Adamus-Grabicka [6*]

[1] Department of Physical Chemistry, Faculty of Chemistry, University of Lodz, Pomorska 163/165, 90-236, Lodz, Poland
[2] University of Lodz Doctoral School of Exact and Natural Sciences, Jana Matejki 21/23, 90-237, Lodz, Poland
[3] Department of Oncobiology and Epigenetics, Institute of Biophysics, Faculty of Biology and Environmental Protection, University of Lodz, Pomorska 141/143, 90-236 Lodz, Poland;
[4] Higher Education and Industrial Cooperation Centre, University of Miskolc, Miskolc-Egyetemváros, 3515, Hungary
[5] Department of the Chemistry of Cosmetic Raw Materials, Faculty of Pharmacy, Medical University of Lodz, Muszynskiego 1, Lodz, 90-151, Poland
6 Department of Medical Chemistry, Faculty of Pharmacy, Medical University of Lodz, Muszynskiego 1, Lodz, 90-151, Poland
* Correspondence: angelika.adamus@umed.lodz.pl;

Connected neighbouring molecules forming motifs that can be described using graph-set notations developed by Margaret C. Etter [10]. Molecule A of asymmetric unit of the crystal structure COMPOUND 1B forming C(9) chain along *b* axis by creating C34A– H34A…O2A^{ii} interaction (**Table S1**, **Figure S1a**). Similar chain forming B molecules along *b* axis (similar to chain A) by C7B – H7B…O2B^{vi} interaction (**Figure S1b**). Molecules B also create $R_2^2(22)$ rings by C35B– H35B…O4B^{iv}, which are responsible for the formation of the mentioned rings (**Figure S1c**).

Table S1. Hydrogen bond geometry in Compound **1B** (Å,°).

D - H...A	D – H	H...A	D...A	D - H...A	Graph-set motifs
C100 – H10B...N36A^{i}	0.98	2.36	2.863(1)	111	D(3)
C36B – H36B...O100^{i}	0.95	2.30	2.958(8)	126	D(3)
C34A – H34A...O2A^{ii}	0.99	2.42	3.350(4)	157	C(9)
C35B – H35B...O4A^{iii}	0.95	2.46	3.174(4)	131	D(3)
C35B – H35B...O4B^{iv}	0.95	2.89	3.659(1)	139	$R_2^2(22)$
C34B – H34D...O4A^{iii}	0.99	2.46	3.278(4)	140	D(3)
C37A – H37A...O1B^{v}	0.95	2.46	3.271(3)	143	D(3)
C7B – H7B...O2B^{vi}	0.95	2.74	3.404(1)	127	C(7)

Symmetry codes: (i) x,y,z; (ii) 5/2-x,1/2+y,3/2-z; (iii) 1-x,1-y,1-z; (iv) -x,2-y,1-z; (v) 1/2-x,-1/2+y,3/2-z; (vi) 1/2-x,-1/2+y,3/2-z;

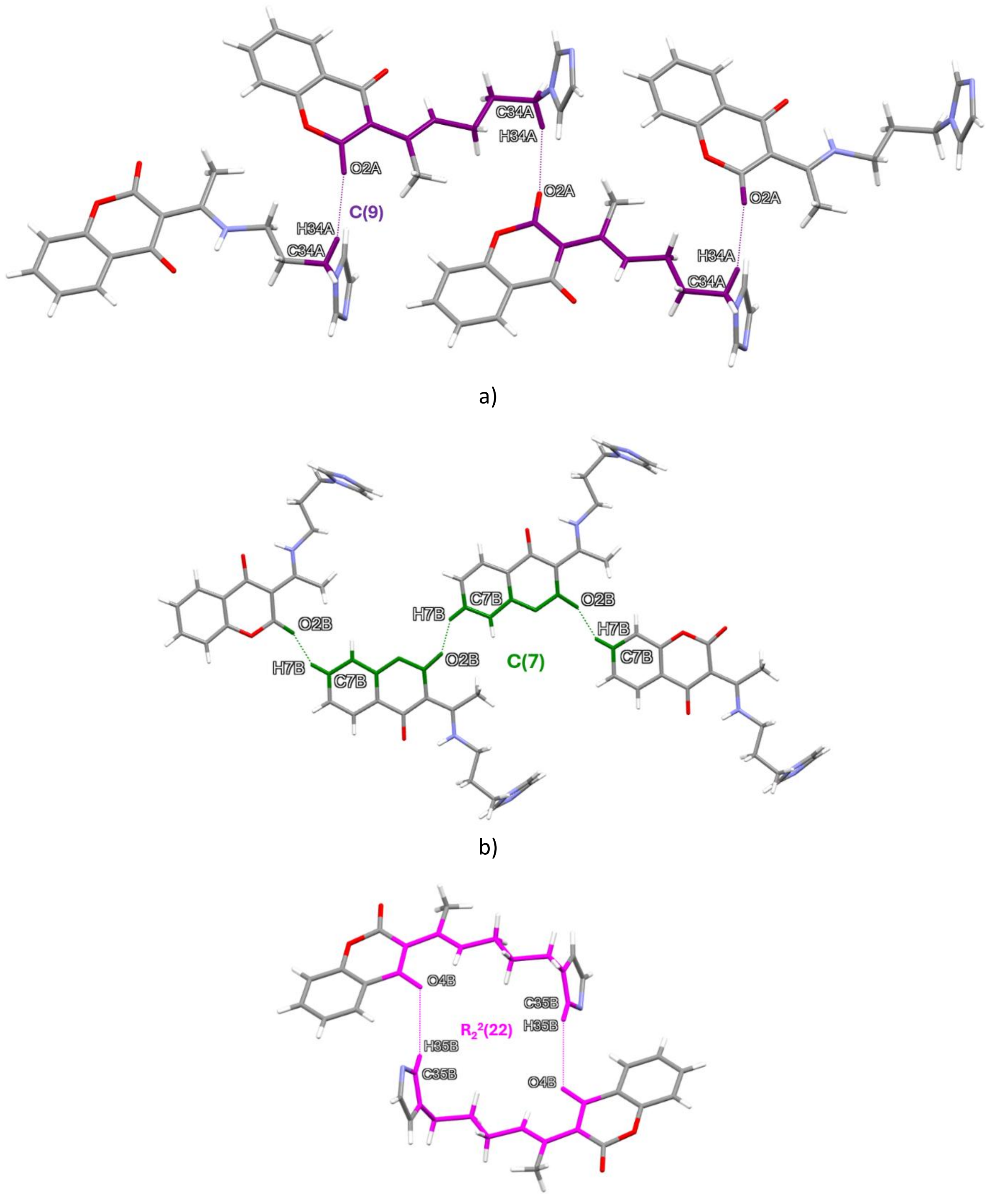

Figure S1. Structural motifs (chains and ring) observed in the crystal structure of Compound **1B**.

All described chains and rings are indirectly linked by methanol molecules forming two C100 – H10B…N36A^{i} and C36B– H36B…O100^{i} interactions described as D(3) dimers (**Figure SS2a**). The remaining three C35B– H35B…O4A^{iii}, C34B– H34D…O4A^{iii} and C37A– H37A…O1B^{v} interactions additionally stabilize crystal structure of Compound **1B** by linking neighbouring chains ang rings what is visualised as dimers D(3) in **Figure S2b-c**. As a result, the molecules form planes that stacked one on top of the other, creating AAA crystal packing arrangement (**Figure S3**).

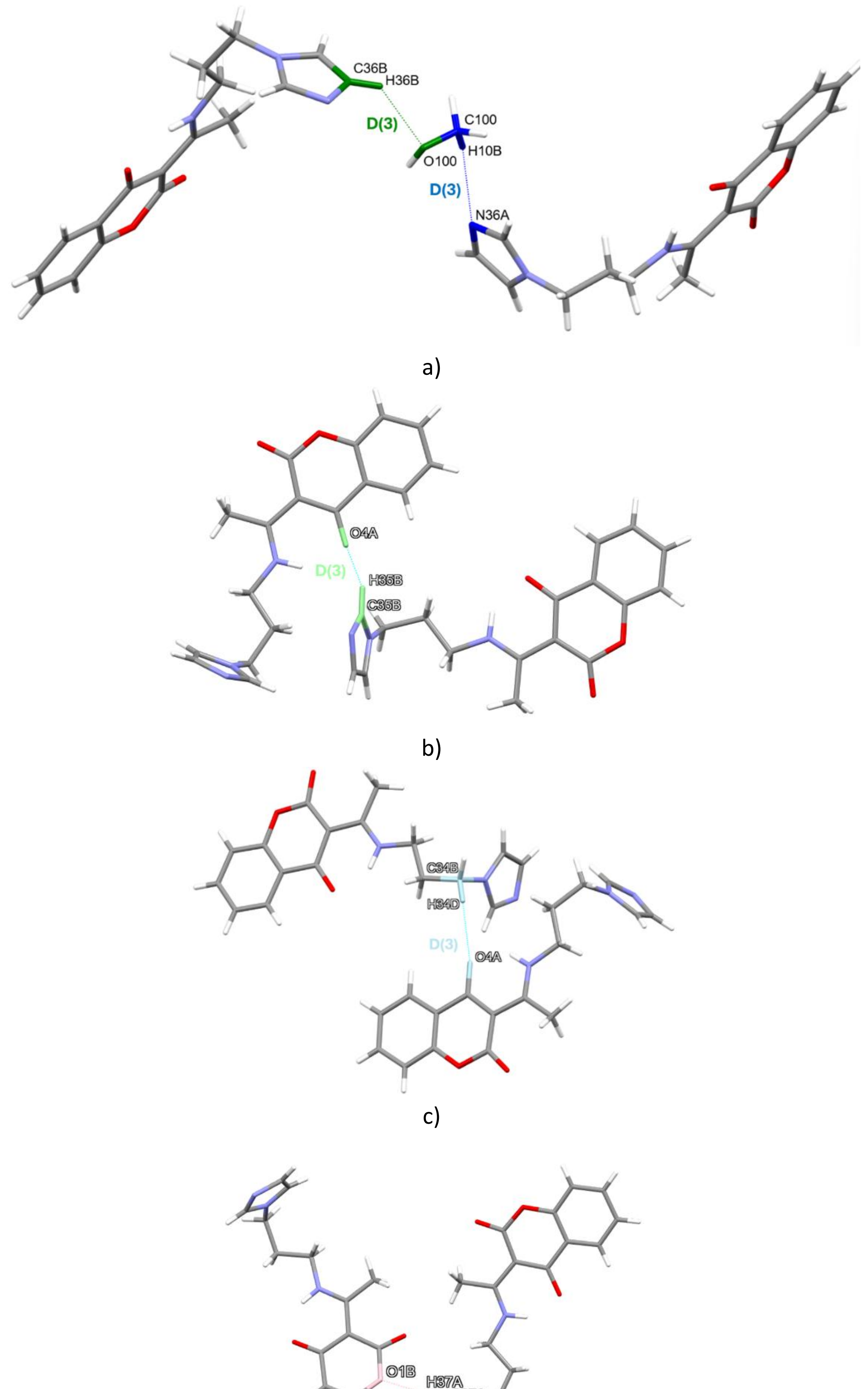

Figure S2. Structural motifs (dimers) observed in the crystal structure of Compound **1B.**

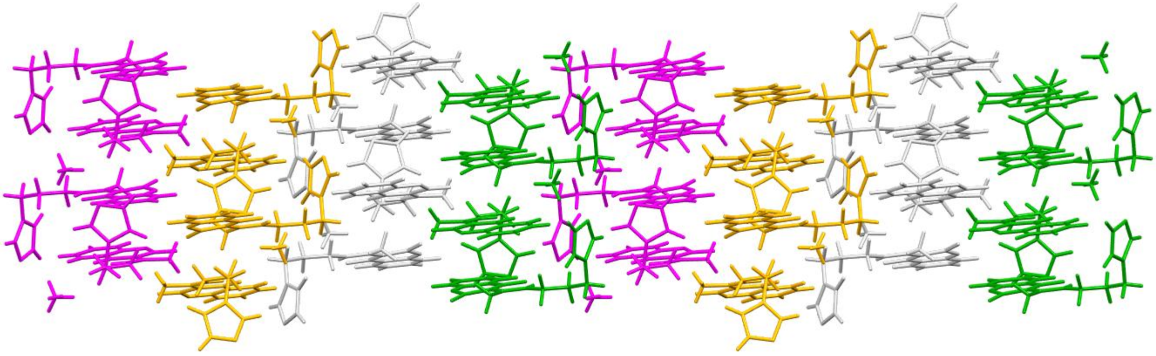

Figure S3. Overall arrangement of molecules observed in the crystal structure of Compound **1B.**

In the crystal structure of Compound **2B** neighbouring molecules are connected forming three types of chain by creating one C13 – H13...N37^{i} interaction and two C33 – H33B...O2^{ii} and C34 – H34B...O2^{ii} interactions, which can be described using graph-set notation as C(13), C(8) and C(9), respectively along [101], *b* and *b* axis (**Table S2**, **Figure S3.**). Subsequent molecules stacked one above the other according to ABAB scheme (**Figure S4.**).

Table S2. Hydrogen bond geometry in Compound **2B** (Å,º).

D - H...A	D – H	H...A	D...A	D - H...A	Graph-set motifs
C13 – H13...N37^{i}	0.95	2.53	3.444(1)	161	C(13)
C33 – H33B...O2^{ii}	0.99	2.56	3.241(1)	126	C(8)
C34 – H34B...O2^{ii}	0.99	2.56	3.173(1)	120	C(9)

Symmetry codes: (i) 3/2+x,3/2-y,1/2+z; (ii) 3/2-x,1/2+y,3/2-z;

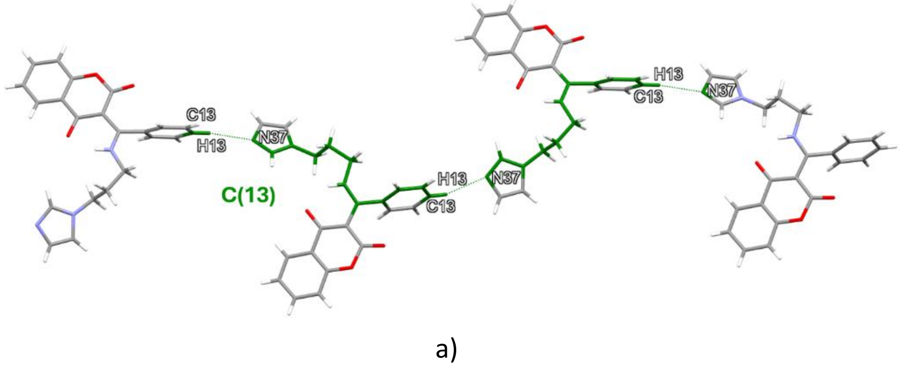

a)

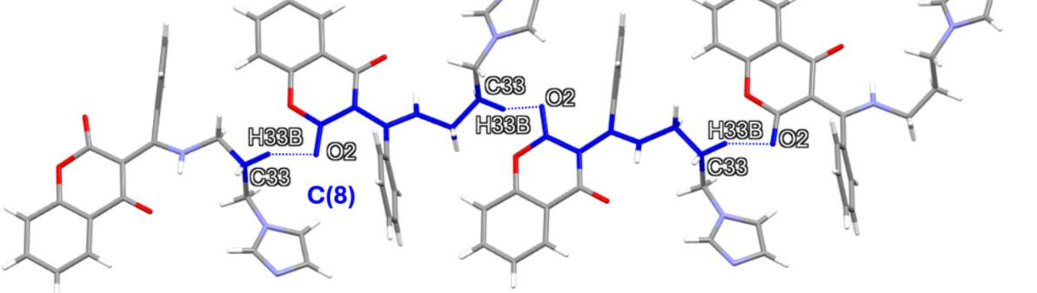

b)

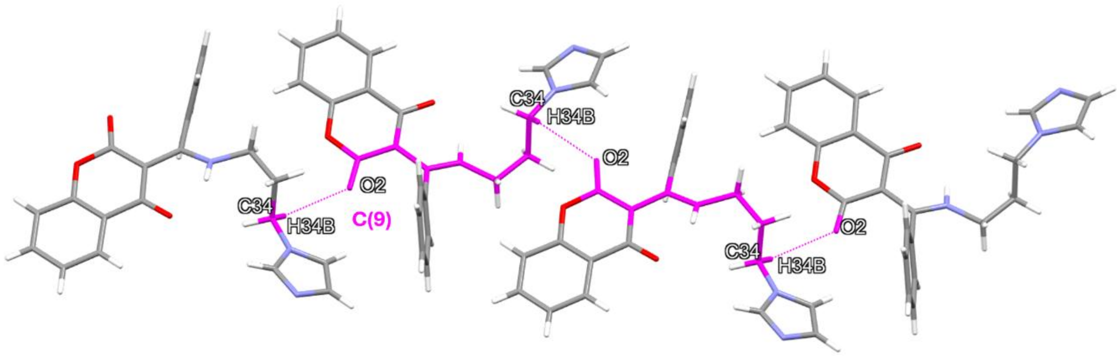

c)

Figure S4. Structural motifs (chains) observed in the crystal structure of Compound **2B**.

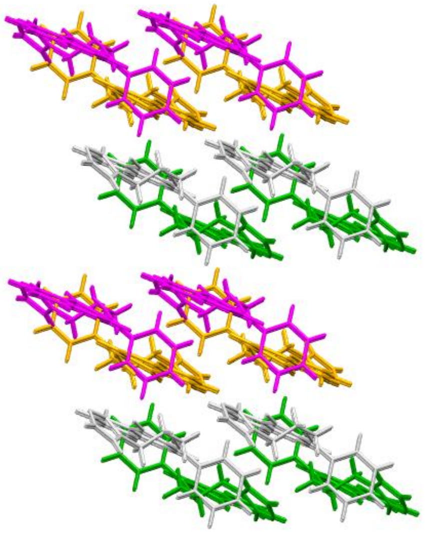

Figure S5. Crystal packing of molecules observed in the crystal structure of Compound **2B**.

In the crystal structure of Compound **1C** neighbouring molecules forming C(13) chain along [101] by creating two O1M – H1M…N38^{i} and C7 – H7…O1M^{ii} interactions using methanol molecule (**Table 3., Figure S6.**). Solvent molecules also participate in the formation D(3) dimer by creating N37 – H37…O1M^{iii}. Another C(6) and C(8) chains are observed between molecules connected by C1 – H1A…O4^{v} along *c* axis and C33 – H33B…O2^{vi} along *b* axis, respectively for C(6) and C(8). Similar to compound Compound **2B**, molecules are arranged in the crystal lattice forming ABAB arrangement (**Figure S7.**).

Table S3. Hydrogen bond geometry in COMPOUND 1C (Å, º)

D - H...A	D – H	H...A	D...A	D - H...A	Graph-set motifs
O1M – H1M...N38^{i}	0.84	1.92	2.755(1)	171	C(13)
C7 – H7...O1M^{ii}	0.95	2.50	3.420(2)	162	

N37 – H37...O1M^{iii}	0.88	1.91	2.789(1)	172	D(3)
C1 – H1A...O2^{iv}	0.98	2.59	3.239(1)	123	$R_2^2(12)$
C1 – H1A...O4^{v}	0.98	2.58	3.305(2)	130	C(6)
C33 – H33B...O2^{vi}	0.99	2.56	3.309(2)	133	C(8)

Symmetry codes: (i) x,y,z; (ii) -1+x,3/2-y,-1/2+z; (iii) 2-x,2-y,2-z; (iv) 1-x,1-y,-z; (v) x,3/2-y,-1/2+z; (vi) 1-x,1/2+y,1/2-z;

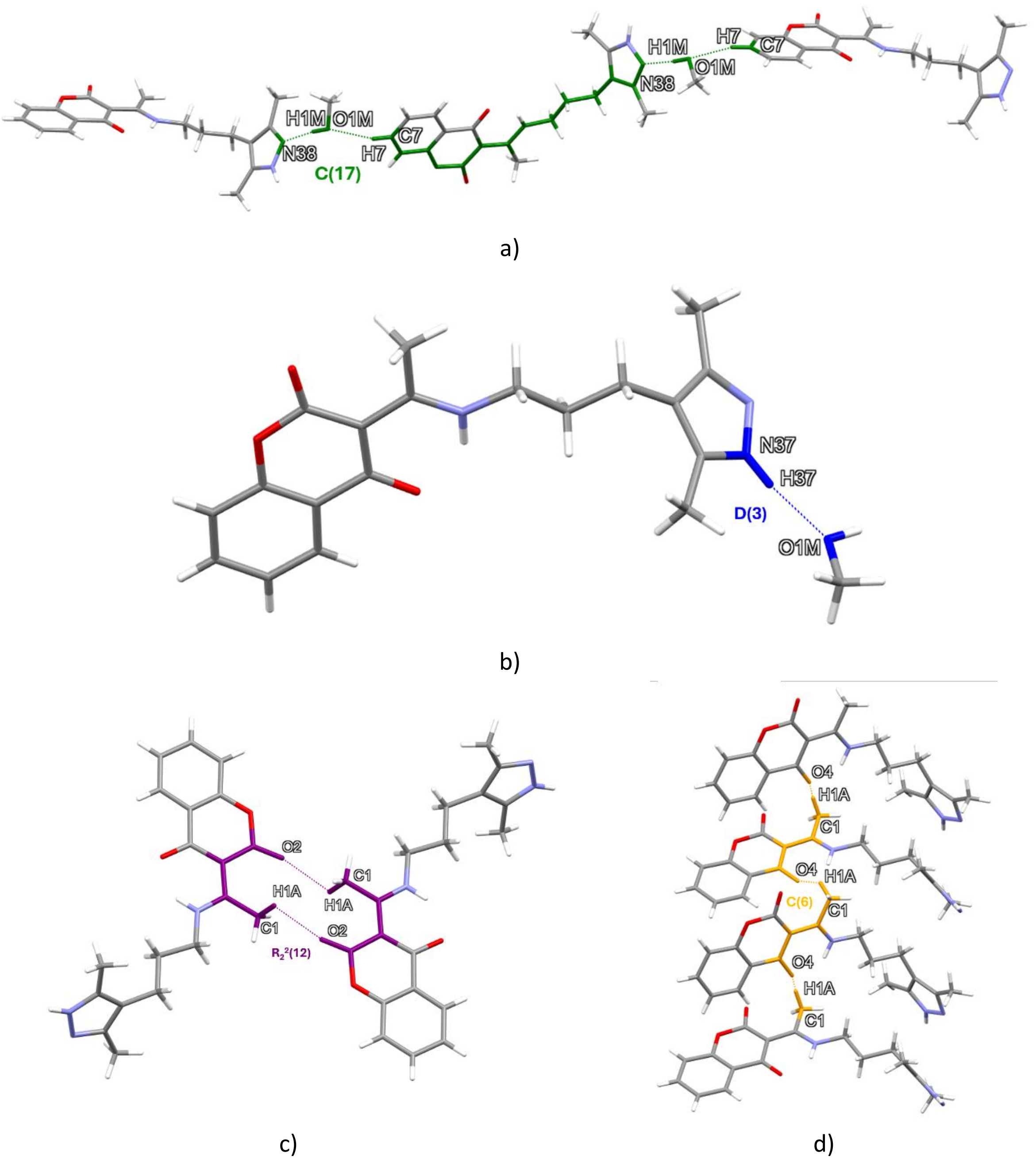

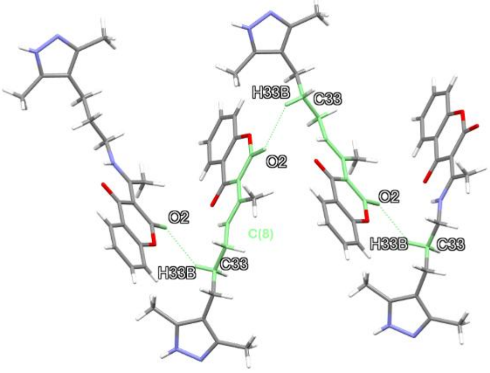

e)

Figure S6. Structural motifs observed in the crystal structure of Compound **1C**.

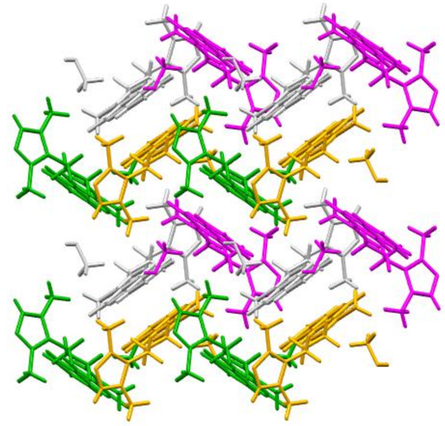

Figure S7. Crystal packing of molecules observed in the crystal structure of Compound **1C**.

In the crystal structure of compound Compound **1D**, the main contribution is accounted for ring motif formed by C1 – H1A...N37^{i}, C8 – H8...O2^{ii} and C34 – H34A...O4^{iii} interactions, respectively described as $R_2^2(20)$, $R_2^2(12)$ and $R_2^2(18)$ using graph-set notations (**Table S4., Figure S8.**). Neighbouring molecules also connect together creating C(9) and C(11) chains formed by C34 – H34B...O2^{iv} and C35 – H35...O2^{v} interactions along [1-10] and *b* axis, respectively. In the crystal structure of Compound **1D** molecules are arranged according ABAB scheme (**Figure S9.**).

Table S4. Hydrogen bond geometry in Compound **1D** (Å,º).

D - H...A	D – H	H...A	D...A	D - H...A	Graph-set motifs
C1 – H1A...N37^{i}	0.98	2.61	3.559(1)	162	$R_2^2(20)$
C8 – H8...O2^{ii}	0.95	2.52	3.395(9)	153	$R_2^2(12)$
C34 – H34A...O4^{iii}	0.99	2.35	3.253(9)	152	$R_2^2(18)$
C34 – H34B...O2^{iv}	0.99	2.58	3.370(1)	136	C(9)
C35 – H35...O2^{v}	0.95	2.35	3.297(1)	174	C(11)

Symmetry codes: (i) 1-x,-y,2-z; (ii) -x,2-y,1-z; (iii) 1-x,-y,1-z; (iv) 1+x,-1+y,z; (v) x,-1+y,z;

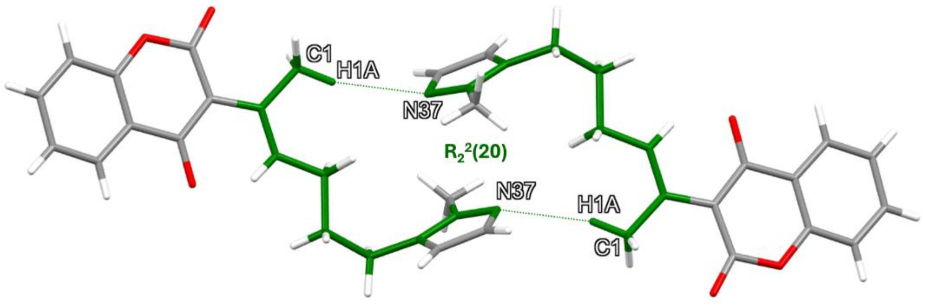
C1
H1A
N37
R22(20)
N37
H1A
C1

a)

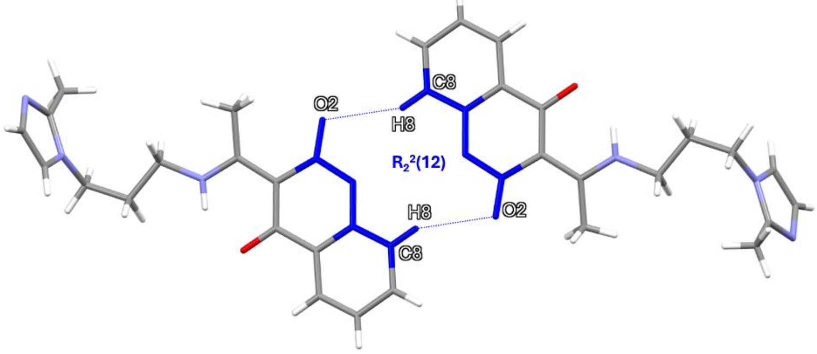
C8
O2
H8
R22(12)
H8
O2
C8

b)

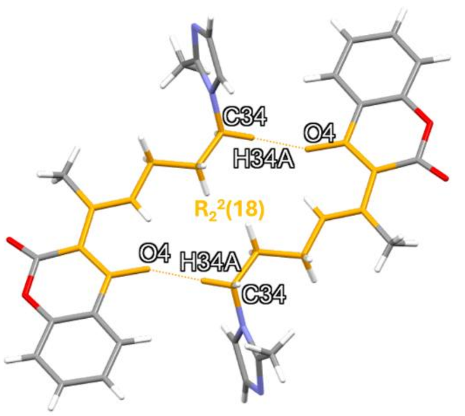
C34
O4
H34A
R22(18)
O4
H34A
C34

c)

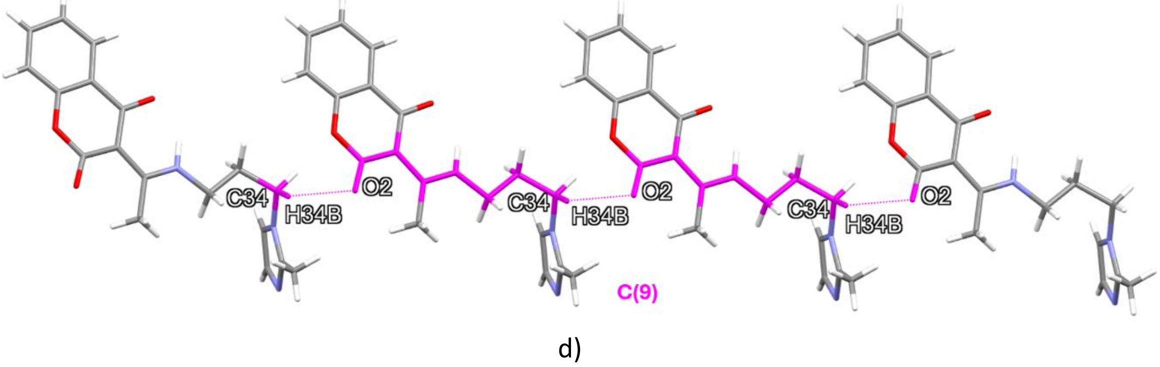
C34
O2
H34B
C34
H34B
O2
C34
H34B
O2
C(9)

d)

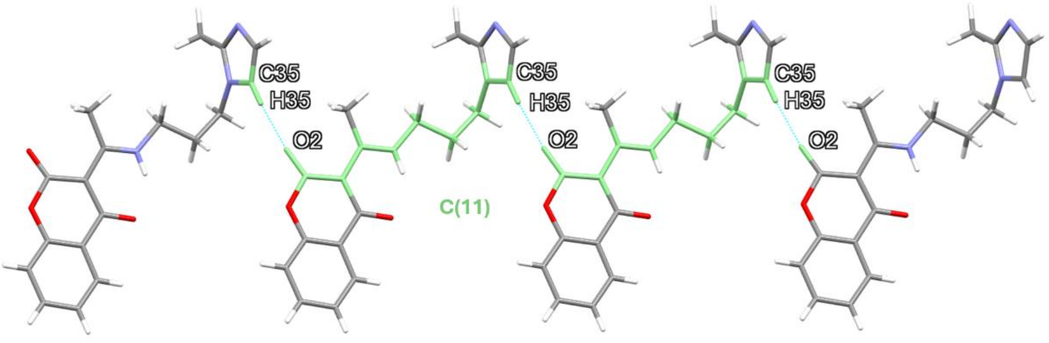

e)

Figure S8. Structural motifs observed in the crystal structure of Compound **1D**.

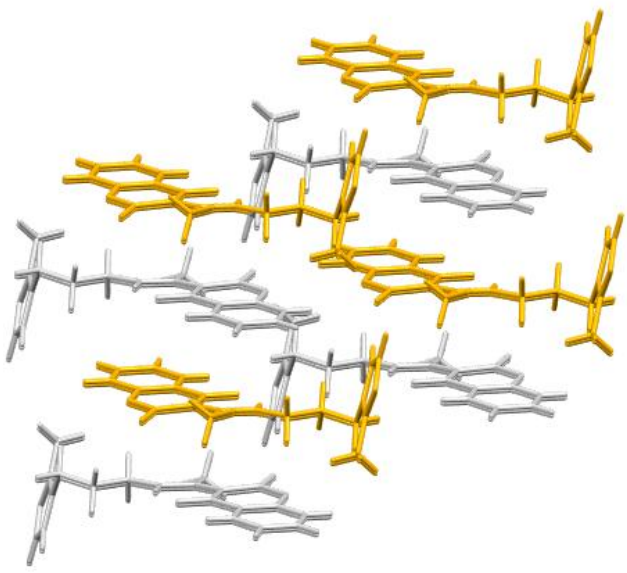

Figure S9. Crystal packing of molecules in the crystal structure of Compound **1D**.

In the crystal structure of Compound **1E** there is observed only chain motif, created by intermolecular interactions in two ways: directly or indirectly by water molecules incorporated in the crystal lattice. Four C6 – H6...O2^{iii}, C7 – H7...N38^{iv}, C39 – H39...O4^{vii} and C40 – H40...O2^{viii} form C(8), C(15), C(13) and C(12) chains directly, respectively along *b* axis, [-101], *b* axis and *c* axis (**Table S5., Figure S10.**). Crystal lattice of this compound is additionally stabilize using water molecules, in which oxygen O1W atom can act as donor (O1W – H1WA...N38^{i}, O1W – H1WB...O2^{ii}) forming C(15) along [-101] or acceptor (C34 – H34B...O1W^{v}, C37 – H37...O1W^{vi}) of hydrogen bonds creating C(7) along *b* axis. All described intermolecular interactions stabilize crystal lattice and arrange crystal architecture according to AAA scheme (**Figure S11.**).

Table S5. Hydrogen bond geometry in Compound **1E** (Å,º).

D - H...A	D – H	H...A	D...A	D - H...A	Graph-set motifs
O1W – H1WA...N38^{i}	0.87	2.01	2.878(2)	175	C(15)
O1W – H1WB...O2^{ii}	0.87	2.09	2.946(2)	169	
C6 – H6...O2^{iii}	0.95	2.46	3.350(2)	157	C(8)

C7 – H7...N38^{iv}	0.95	2.59	3.413(2)	145	C(15)
C34 – H34B...O1W^{v}	0.99	2.51	3.490(2)	170	C(7)
C37 – H37...O1W^{vi}	0.95	2.56	3.421(2)	150	
C39 – H39...O4^{vii}	0.95	2.57	3.487(2)	164	C(13)
C40 – H40...O2^{viii}	0.95	2.52	3.343(2)	145	C(12)

Symmetry codes: (i) x,y,z; (ii) -1+x,1/2-y,1/2+z; (iii) x,1+y,z; (iv) 1+x,3/2-y,-1/2+z; (v) 1+x,y,z; (vi) -x,1/2+y,3/2-z; (vii) 1-x,-1/2+y,3/2-z; (viii) x,1/2-y,1/2+z;

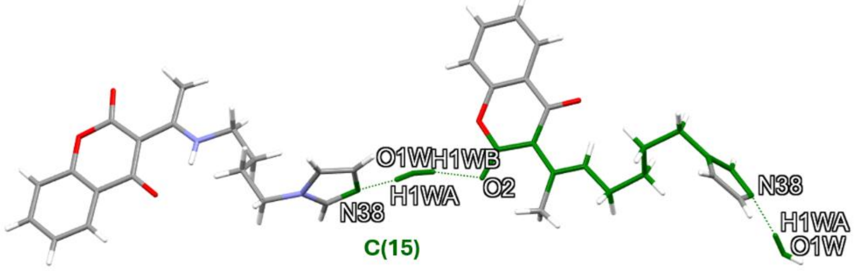

a)

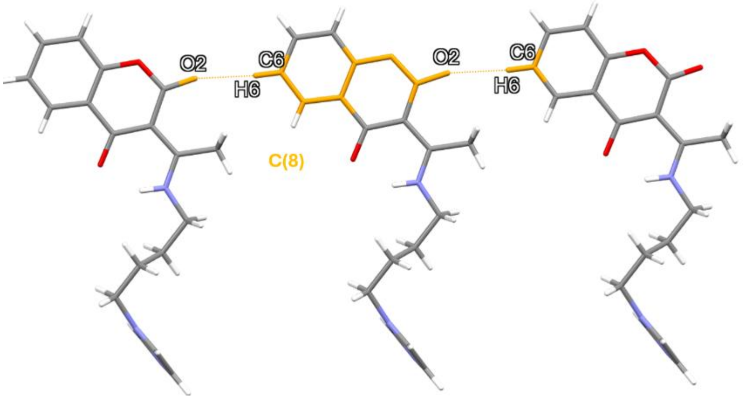

b)

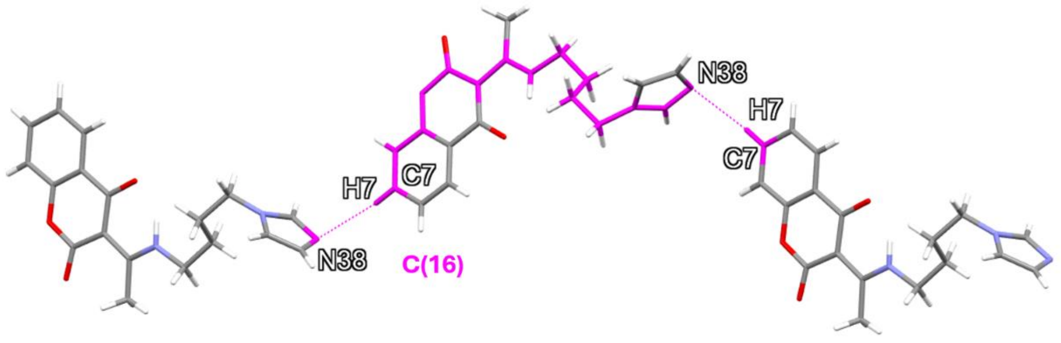

c)

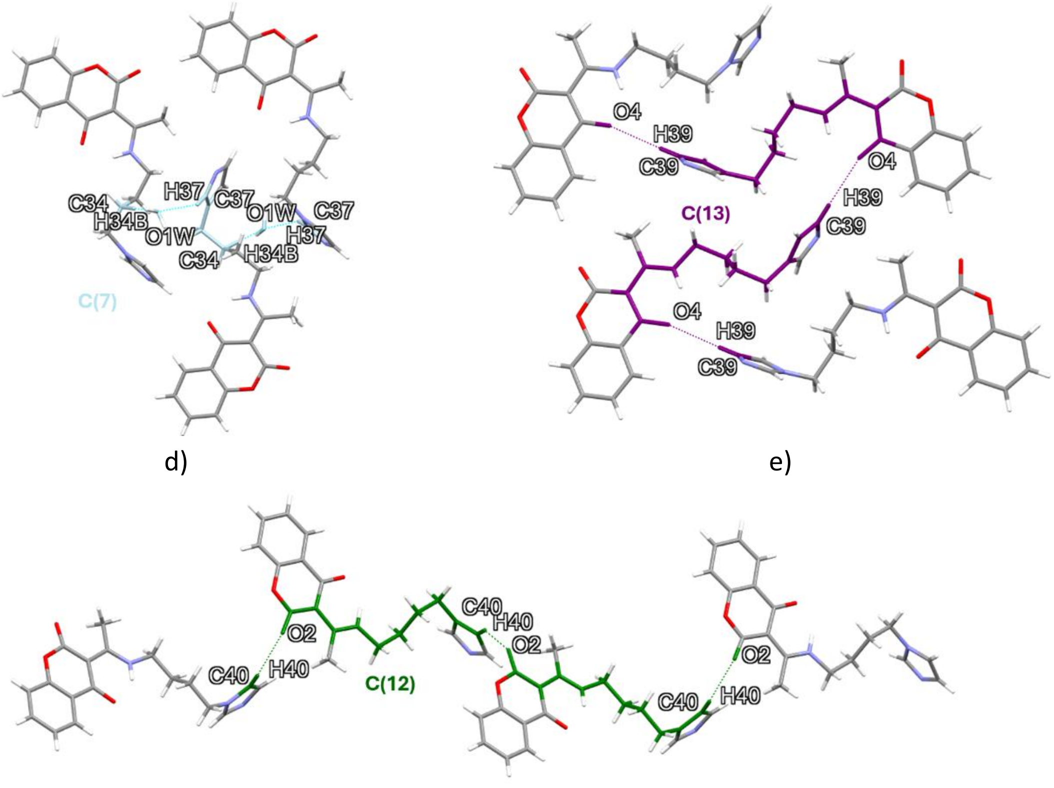

d) e)

f)

Figure S10. Structural motifs observed in the crystal structure of Compound **1E.**

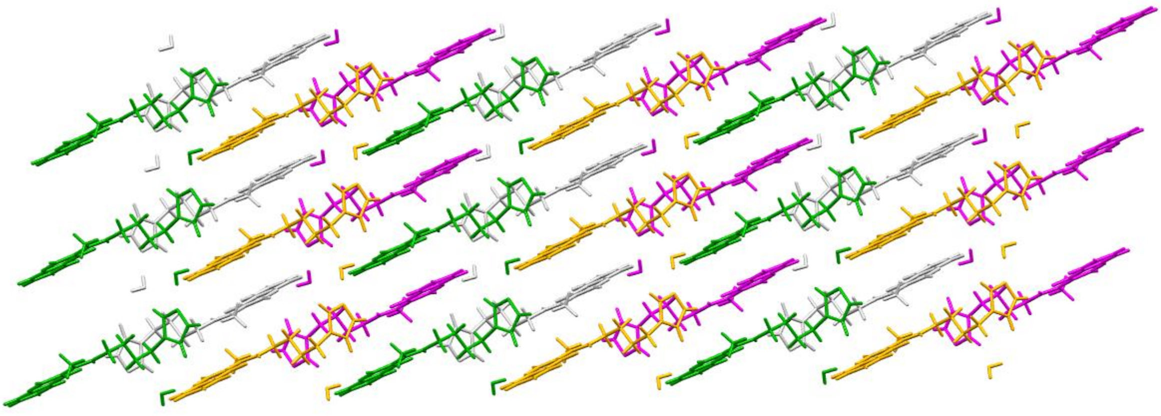

Figure S11. Crystal packing of molecules in the crystal structure of Compound **1E**.

In the crystal structure of Compound **1F** there are two chromone molecules and one water molecule. Intermolecular interactions stabilize crystal lattice forming three chains described using graph-set notation as C(21), C(11) and C(11), respectively by creating C34B – H34D...O4A^{iii} and C37A – H37A...O2B^{i} (chain C(21)), C39A – H39A...O2A^{vi} and C39B – H39B...O2B^{i} interactions (**Table S6., Figure S12.**). All described chains are formed along *b* axis. However, C37A – H37A...O2B^{i} and C37B – H37B...O2A^{iv} interactions create $R_2^2(24)$ ring. Additionally, incorporated water molecules in the crystal lattice stabilize crystal structure by forming $R_4^4(26)$ ring, creating O1W – H1WA...O2B^{i} and O1W – H1WB...N36B^{ii} interactions. There is only one dimer motif marked as D(3), created by C38A – H38A...O1W^{v} interaction. In the crystal lattice of Compound **1F**,

molecules are arranged in alternating rows of molecules A and B forming planes (**Figure S13a**), which stacked one above the other according to ABAB scheme (**Figure 13b**).

Table S6. Hydrogen bond geometry in Compound **1F** (Å,º).

D - H...A	D – H	H...A	D...A	D - H...A	Graph-set motifs
O1W – H1WA...O2B^{i}	0.87	2.08	2.928(4)	166	$R_4^4(26)$
O1W – H1WB...N36B^{ii}	0.87	2.10	2.905(4)	154	
C34B – H34D...O4A^{iii}	0.99	2.49	3.357(4)	147	C(21)
C37A – H37A...O2B^{i}	0.95	2.49	3.328(4)	147	
C37B – H37B...O2A^{iv}	0.95	2.47	3.358(5)	155	$R_2^2(24)$
C38A – H38A...O1W^{v}	0.95	2.52	3.343(5)	145	D(3)
C39A – H39A...O2A^{vi}	0.95	2.55	3.435(5)	155	C(11)
C39B – H39B...O2B^{i}	0.95	2.48	3.381(5)	158	C(11)

Symmetry codes: (i) 1/2-x,-1/2+y,1/2-z; (ii) 1/2+x,1/2-y,1/2+z; (iii) -1+x,y,z; (iv) 1/2-x,1/2+y,1/2-z; (v) x,y,z; (vi) 3/2-x,1/2+y,1/2-z;

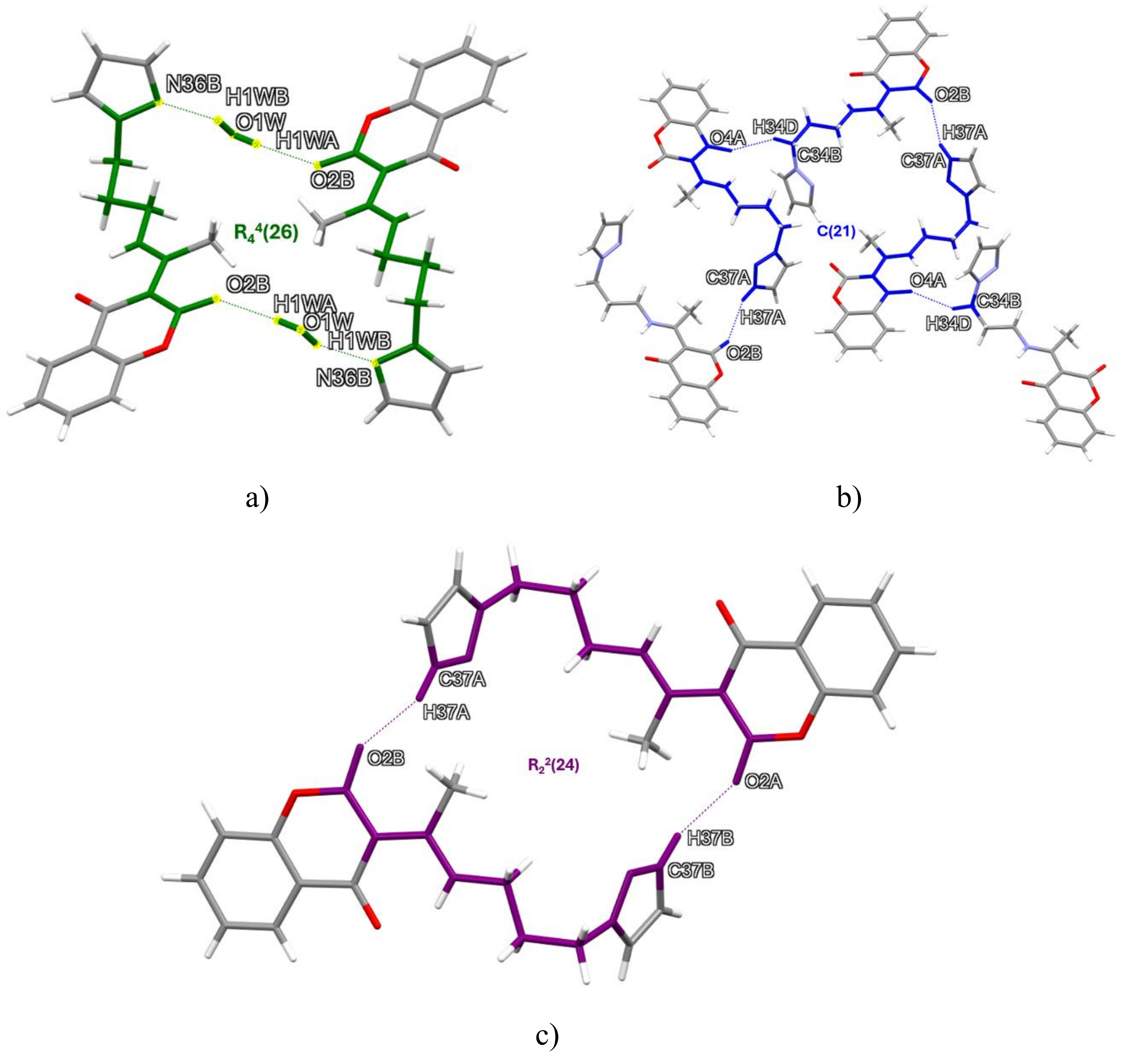

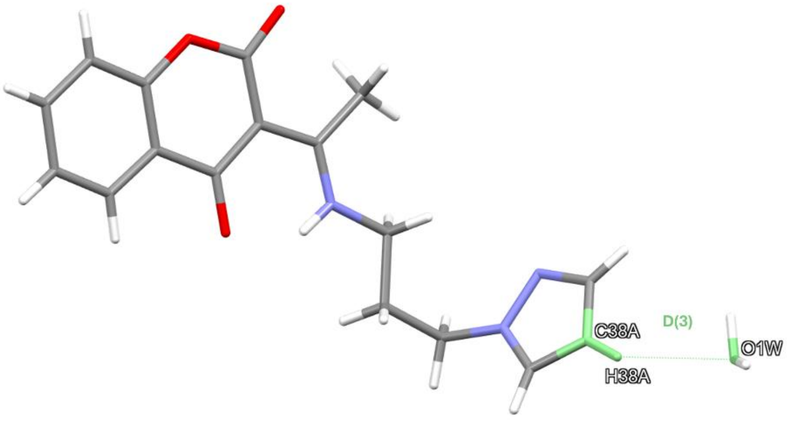

d)

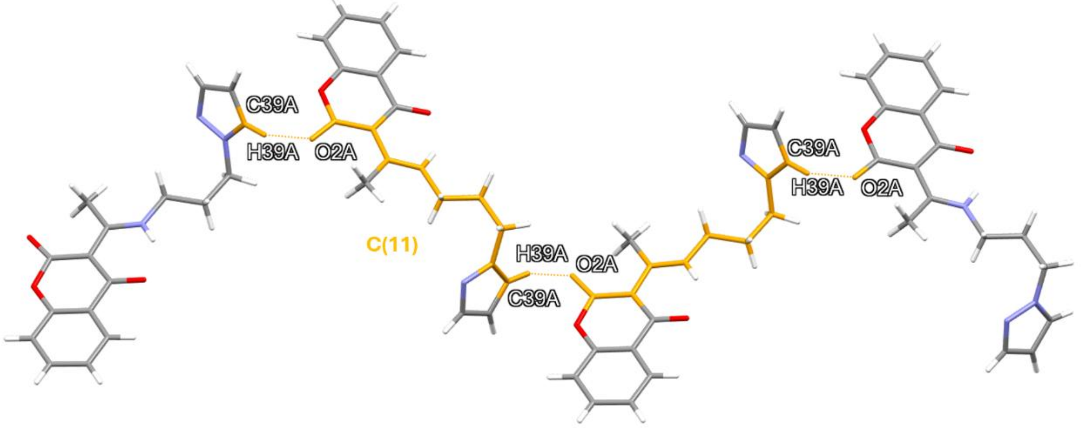

e)

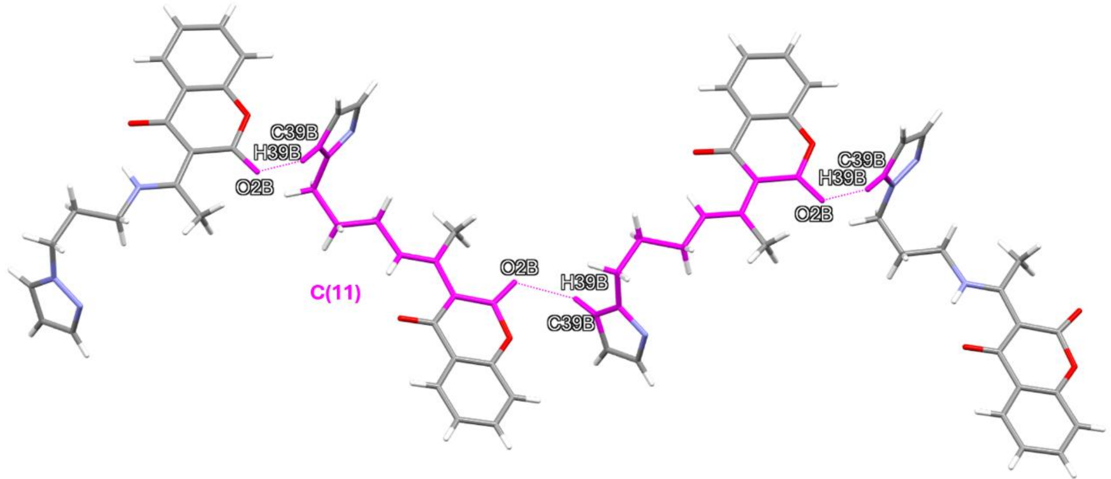

f)

Figure S12. Structural motifs observed in the crystal structure of Compound **1F**.

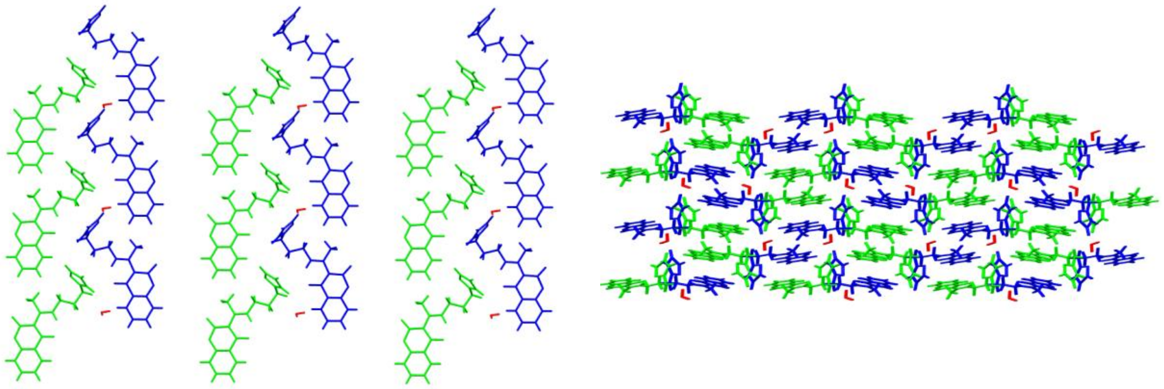

a) b)

Figure S13. Crystal packing of molecules in the crystal structure of Compound **1F**: a) plane, b) layers ABAB

Fig. S14. ITC titration of 0.4 mM chromone ester Compound **1** (in cell) with 10 mM β-CD in DMSO (in syringe) w 25℃.

Thermal power as a function of time for titration of chromone derivatives titration with β-CD and corresponding effects of β-CD dilution in DMSO	Heat effects of chromone derivatives titration with β-CD and corresponding heat effects of β-CD dilution in DMSO	Direct heat effects of chromone derivatives interactions with β-CD
**A) Compound 1**		
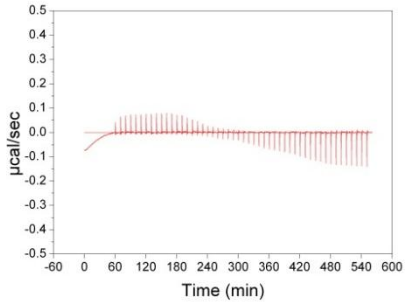 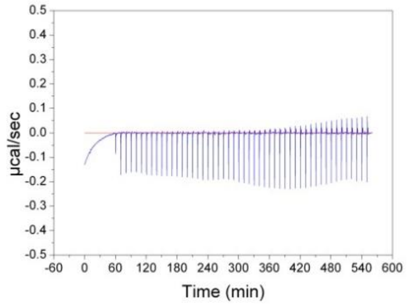	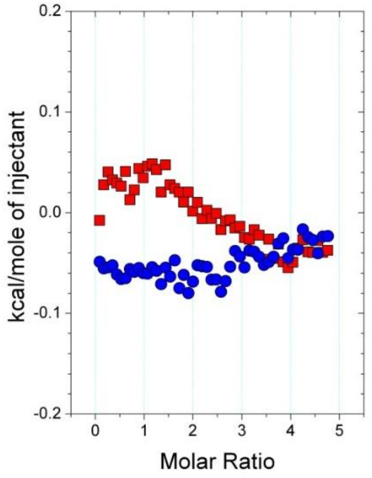	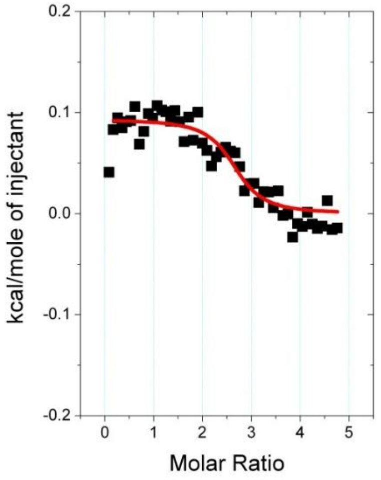

B) Compound 1A

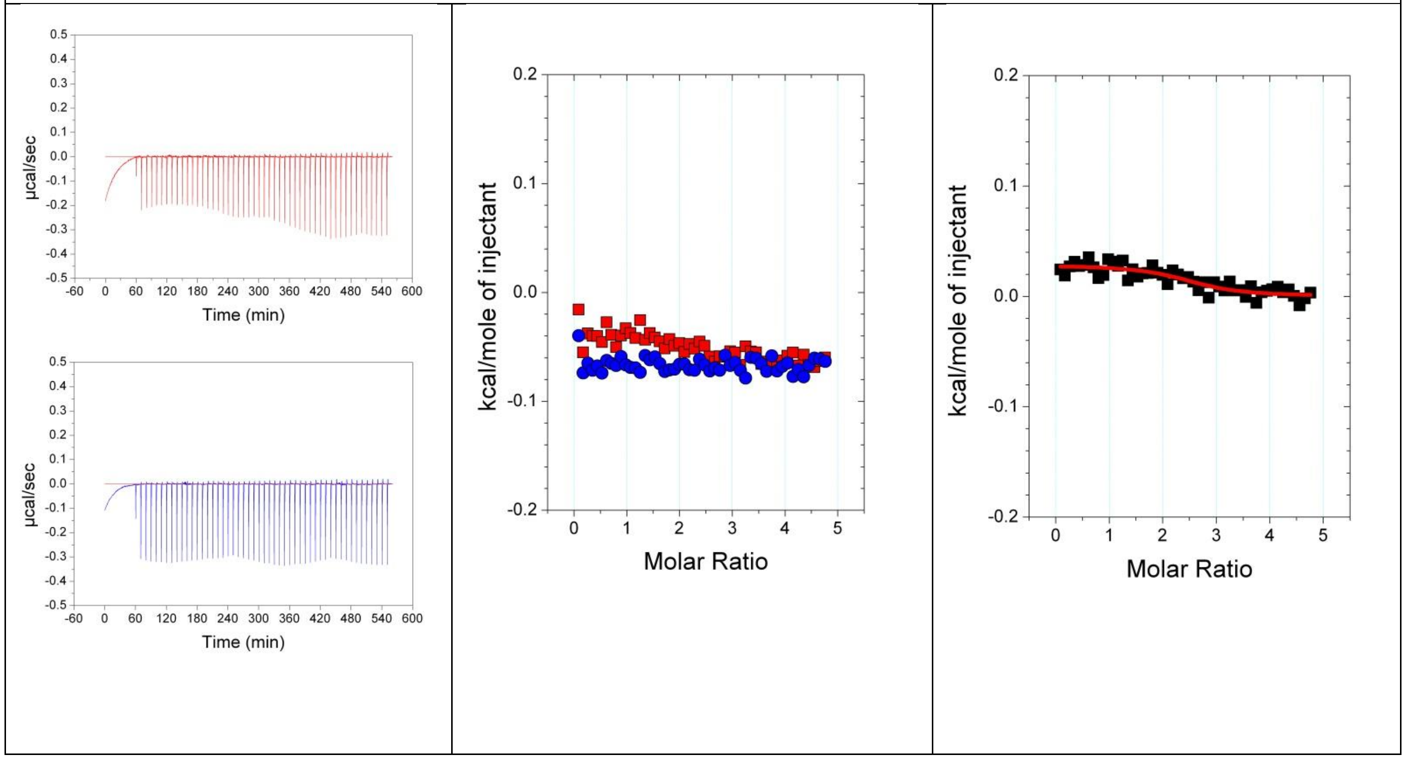

C) Compound 1D

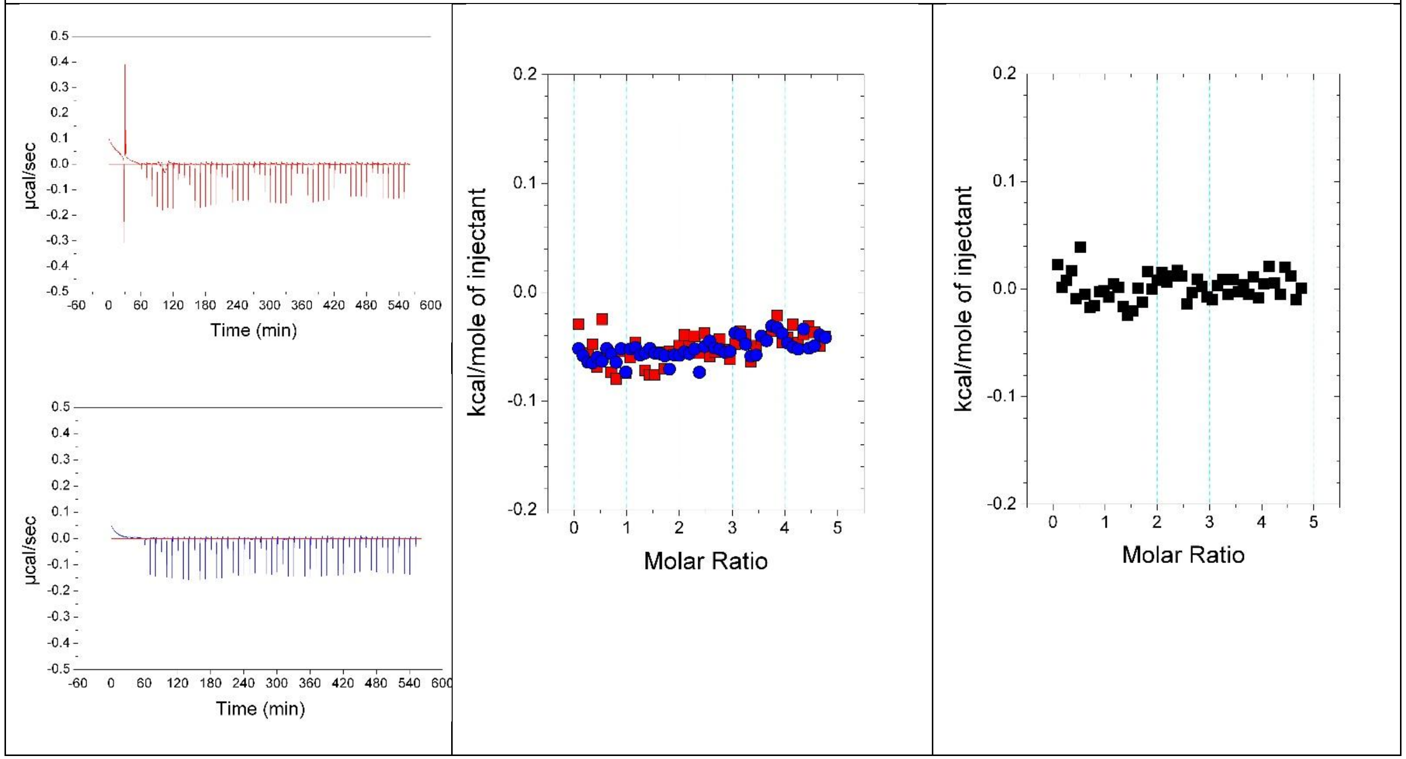

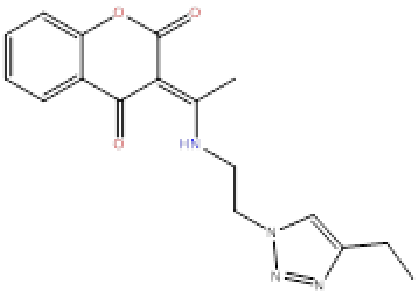

Fig. S15. Structure of Compound **1A**, which was synthetized in our previous work [**SM_R1**].

Table S7. Thermodynamic parameters comparison of the supramolecular complexes formation between β-cyclodextrin and its ligands in DMSO solvent at 25°C.

Guest	n	$logK$	ΔH [kcal mol^{-1}]	ΔS [cal K^{-1} mol^{-1}]	ΔG [kcal mol^{-1}]
**Compound 1**	2.6	4.86	0.093	22.6	–6.4
**Compound 1A**	2.6	4.08	0.028	18.8	–5.6
**3** [**SM_R2**]	1	3.57	–0.259	16.2	–5.1
**3a** [**SM_R2**]	1	3.40	–0.212	15.5	–4.8
**5** [**SM_R2**]	1	3.37	–0.043	15.6	–5.1
**Ezetimibe** [**SM_R3**]	1.1	3.23	–0.160	14.9	–4.5
**Flavanone** [**SM_R4**]	0.9	3.58	–0.026	16.3	–4.9
**4'-Chloroflavanone [SM_R4**]	0.9	3.50	–0.038	15.9	–4.8

Supplementary Material References

[SM_R1] Czapnik, P.; Adamus-Grabicka, A.; Budzisz, E.; Domagała, M.; Małecka, M. Noncovalent Interactions and Crystal Packing Chromono-3-Carboxylic Acid Esters and Their Triazole Derivatives: A Combined Experimental and Theoretical Study. *Cryst. Growth Des.* **2026**, *26*, 1896–1916, doi:10.1021/acs.cgd.5c01113.

[SM_R2] Adamus-Grabicka, A.A.; Hikisz, P.; Stepniak, A.; Malecka, M.; Paneth, P.; Sikora, J.; Budzisz, E. Molecular Pro-Apoptotic Activities of Flavanone Derivatives in Cyclodextrin Complexes: New Implications for Anticancer Therapy. Int. J. Mol. Sci. 2024, 25, 8488, doi:10.3390/ijms25158488.

[SM_R3] Biernacka, M.; Ilyich, T.; Zavodnik, I.; Pałecz, B.; Stepniak, A. Studies of the Formation and Stability of Ezetimibe-cyclodextrin Inclusion Complexes. Int. J. Mol. Sci. 2022, 23, doi:10.3390/ijms23010455.

[SM_R4] Stepniak, A.; Biernacka, M.; Malecka, M.; Palecz, B. Host-Guest Complexes of Flavanone and 4′-Chloroflavanone with Naturals and Modified Cyclodextrin: A Calorimetric and Spectroscopy Investigations. Molecules 2024, 29, 3123, doi:10.3390/molecules29133123.
